# Silencing by H-NS Potentiated the Evolution of *Salmonella*

**DOI:** 10.1371/journal.ppat.1004500

**Published:** 2014-11-06

**Authors:** Sabrina S. Ali, Jeremy Soo, Chitong Rao, Andrea S. Leung, David Hon-Man Ngai, Alexander W. Ensminger, William Wiley Navarre

**Affiliations:** Department of Molecular Genetics, University of Toronto, Toronto, Ontario, Canada; McMaster University, Canada

## Abstract

The bacterial H-NS protein silences expression from sequences with higher AT-content than the host genome and is believed to buffer the fitness consequences associated with foreign gene acquisition. Loss of H-NS results in severe growth defects in *Salmonella*, but the underlying reasons were unclear. An experimental evolution approach was employed to determine which secondary mutations could compensate for the loss of H-NS in *Salmonella*. Six independently derived *S.* Typhimurium *hns* mutant strains were serially passaged for 300 generations prior to whole genome sequencing. Growth rates of all lineages dramatically improved during the course of the experiment. Each of the *hns* mutant lineages acquired missense mutations in the gene encoding the H-NS paralog StpA encoding a poorly understood H-NS paralog, while 5 of the mutant lineages acquired deletions in the genes encoding the *Salmonella* Pathogenicity Island-1 (SPI-1) Type 3 secretion system critical to invoke inflammation. We further demonstrate that SPI-1 misregulation is a primary contributor to the decreased fitness in *Salmonella hns* mutants. Three of the lineages acquired additional loss of function mutations in the PhoPQ virulence regulatory system. Similarly passaged wild type *Salmonella* lineages did not acquire these mutations. The *stpA* missense mutations arose in the oligomerization domain and generated proteins that could compensate for the loss of H-NS to varying degrees. StpA variants most able to functionally substitute for H-NS displayed altered DNA binding and oligomerization properties that resembled those of H-NS. These findings indicate that H-NS was central to the evolution of the Salmonellae by buffering the negative fitness consequences caused by the secretion system that is the defining characteristic of the species.

## Author Summary

H-NS is an abundant DNA-binding protein found in enteric bacteria including the important pathogens *Escherichia*, *Salmonella*, *Vibrio*, and *Yersinia*, that plays a primary role in defending the bacterial genome by silencing AT-rich foreign genes. H-NS has been hypothesized to facilitate the evolution of bacterial species by acting as a buffer against the negative consequences that can occur when new genes are incorporated into pre-existing genetic landscapes. Here experimental evolution and whole-genome sequencing were employed to determine the factors underlying the severe growth defects displayed by *Salmonella* strains lacking H-NS. Through tracking the evolution of several independently derived mutant lineages, we find that compensatory mutations arise quickly and that they occur in loci related to virulence. A frequent outcome was loss of the *Salmonella* Pathogenicity Island-1, the defining genetic island of the genus *Salmonella*. Among other things these findings demonstrate that H-NS has enabled the birth of a new and important bacterial pathogen by buffering the fitness consequences caused by overexpression of SPI-1. These findings are likely generalizable to pathogens such as *E. coli*, *Yersinia*, *Shigella*, and *Vibrio cholerae*, all of which maintain a pool of “expensive” AT-rich virulence genes that are repressed by H-NS.

## Introduction

Horizontal gene transfer (HGT) has profoundly shaped the course of bacterial speciation and diversification. The uptake of ‘pre-assembled’ genetic loci involved in antibiotic resistance, virulence, phage resistance or novel modes of metabolism can instantly confer beneficial phenotypes to the recipient cell. HGT events have been critical in the evolution of almost all bacterial pathogens from their non-pathogenic progenitors [1]–[5]. Two of the critical events when the Salmonellae diverged from their last common ancestor with *E. coli* were the acquisition of the *Salmonella* Pathogenicity Island-1 (SPI-1) and the tetrathionate reductase *ttr* gene clusters [6]–[8]. SPI-1 is a 40 kb genomic island encoding a Type 3 Secretion System (TTSS) required for triggering inflammation and for invasion of cells lining the intestinal mucosa [9]–[12]. Together these systems enable *Salmonella* to outcompete other microbes in the mammalian gut where SPI-1 induces a potent oxidative inflammation that generates tetrathionate, which then serves as a terminal electron acceptor for anaerobic respiration that is available solely to *Salmonella* but not other gut microbes [7].

Despite its overall importance to bacterial evolution, any individual HGT event is more likely to reduce bacterial fitness than to improve it. Even potentially beneficial genes can disrupt regulatory networks or drain metabolic resources away from the production of energy or biomass if they are not properly regulated [13]. Indeed, studies examining the barriers to new gene acquisition found that genes expressed at high levels are much more likely to be selected against in the new host [14], [15]. Virulence-associated genes, including those that encode secretion systems like the TTSS, can be particularly costly and are often lost in the absence of purifying selection (e.g. virulence attenuation by laboratory passage) [16]–[19]. For example, triggering TTSS activation from the *Shigella* virulence plasmid in liquid media causes the destabilization and eventual loss of the plasmid from the population [20].

The nucleoid associated protein H-NS was proposed to buffer the fitness costs associated with HGT by silencing genes with a %GC content significantly lower than the host genome average and are therefore likely to have been acquired from a foreign source [21]–[25]. H-NS confers this benefit both by counteracting transcription at standard promoters and by preventing spurious transcription within an adenine and thymine-rich (AT-rich) open reading frame at sequences that can adventitiously resemble a bacterial promoter [26]. H-NS exhibits low sequence specificity and targets DNA by recognizing specific structural features in the minor grove of AT-rich DNA [27], [28]. H-NS polymerizes along target AT-rich sequences by virtue of two independent dimerization domains, leading to the formation of extended nucleoprotein filaments [29]–[32]. As a result of its activity, H-NS regulates the majority of horizontally acquired sequences in species such as *E. coli*, *Yersinia*, *Shigella* and *Salmonella* [1], [33]–[35].

Members of the H-NS protein family are distributed between the alpha, beta and gamma proteobacteria. Functional analogues that bear minimal sequence or structural resemblance to H-NS have been identified in *Pseudomonas sp.* (MvaT and MvaU) and *Mycobacteria sp.* (Lsr2) [36], [37]. While global gene expression data sets from *Escherchia coli* (*E. coli*), *Yersinia enterolitica* (*Y. enterolitica*), *Salmonella enterica* Sv. Typhimurium (*S.* Typhimurium), *Pseudomonas aeruginosa* (*P. aeruginosa*) and *Mycobacteria smegmatis* (*M. smegmatis*) point to a common role for the H-NS/MvaT/Lsr2 proteins as silencers of foreign AT-rich sequences, the fitness consequences of mutating the xenogeneic silencers among these species differs significantly [21]–[24], [38]–[40]. In *P. aeruginosa*, MvaT and MvaU together are essential and depletion of both of these proteins results in the activation of the Pf4 prophage, which kills the bacterial cell [41]. In most strains of *E. coli*, mutations in *hns* mildly impede growth rates whereas failed attempts at constructing *hns* mutants in *Y. enterolitica* and *Y. pseudotuberculosis* strains strongly suggest *hns* is an essential gene in *Yersinia sp.* [42], [43]. *S.* Typhimurium strain 14028s *hns* mutants are only viable if additional mutations are present in either the PhoP-PhoQ two component signaling system or the stationary phase sigma factor RpoS [22]. What remains unclear is why global H-NS mediated gene silencing is so critical for the fitness of *S.* Typhimurium and *Y. enterolitica*, but is largely dispensable to other closely related species such as *E. coli*.

Several members of the *Enterobacteriaceae* including *E. coli*, *S.* Typhimurium and *Shigella flexneri* (*S. flexneri*) encode a second H-NS-like protein, StpA. StpA shares 53% sequence identity with H-NS as well as several functional properties, such as the ability to self-associate and bind AT-rich DNA [44]–[48]. H-NS and StpA also share a similar domain architecture exemplified by the detection of StpA/H-NS heterodimers *in vivo* and *in vitro* [49]–[52]. Global transcript analysis and ChIP-on-chip data sets indicate StpA and H-NS co-localize in *E. coli* and *S.* Typhimurium, but the loss of *stpA* only affects the transcript levels of a subset of these loci [47], [48]. In fact, loss of StpA alone does not generate observable phenotypes but will further impair the fitness of strains lacking H-NS [45], [53], [54]. The mild effects of *stpA* depletion may be attributed to low intracellular StpA concentrations [46], [55]. StpA is a substrate of the Lon protease and a StpA point mutation, F21C, that imparts resistance to proteolytic cleavage also restored stationary phase viability to an *E. coli hns* mutant strain [56]. Other reports, however, suggest H-NS and StpA exhibit similar expression levels with the StpA protein reaching 25 000 copies per cell at mid-exponential phase and H-NS reaching 20 000 copies [57]. Despite significant sequence homology between H-NS and StpA, the basis for their functional dissimilarities remains unknown.

In this study, we employed an experimental evolution strategy to select for mutations that compensate for the strong fitness defects of *S.* Typhimurium *hns* mutants. Using whole genome sequencing we identified parallel adaptations in many of the *hns* mutant lineages including genomic deletions in the pathogenicity locus SPI-1 and non-synonomous changes in the gene encoding StpA. The *stpA* mutations altered residues in the oligomerization domain and several enhanced the ability of StpA to silence *hns* regulated genes without having an effect on StpA expression levels. Much of the fitness defect in the *hns* mutants could be attributed to overexpression of SPI-1. This work provides compelling evidence that H-NS potentiates bacterial speciation by improving bacterial tolerance for horizontally acquired sequences. These findings also suggest that fitness-cost buffering by xenogeneic silencing proteins contributes to the observed tendency for genomic islands to be AT-rich.

## Results

### Passaging of *S.* Typhimurium *hns* mutants for 30 days leads to strains with improved fitness

Disruption of the *hns* gene in the wild type *S.* Typhimurium 14028s strain background severely restricts its growth rate to the point where cultivation is difficult [22]. However, we previously demonstrated that *hns* mutations can be achieved in strains that harbor additional mutations in the gene encoding the alternative sigma factor RpoS (σ^S^ or σ^38^). Alleles that reduce σ^S^ activity frequently arise during laboratory passage and are present in another commonly used *Salmonella* laboratory strain, LT2. The alleviating effect of *rpoS* mutations in the *hns* mutants may be due to the fact that loss of H-NS dramatically improves the stability of RpoS [58], which may cause the inappropriate overexpression of stationary-phase genes and interfere with the expression of housekeeping genes controlled by RpoD. To facilitate this study the *hns* gene from *S.* Typhimurium 14028s was replaced with a kanamycin resistance cassette in a background harboring a 5 amino acid in frame deletion within the coding region of *rpoS* that reduces RpoS activity (referred to as rpoS*) [22]. Although this additional mutation improved the tolerance of 14028s for *hns* mutations, Δhns/rpoS* strains continue to display severe growth defects including dramatically reduced colony size.

**Figure 1:**
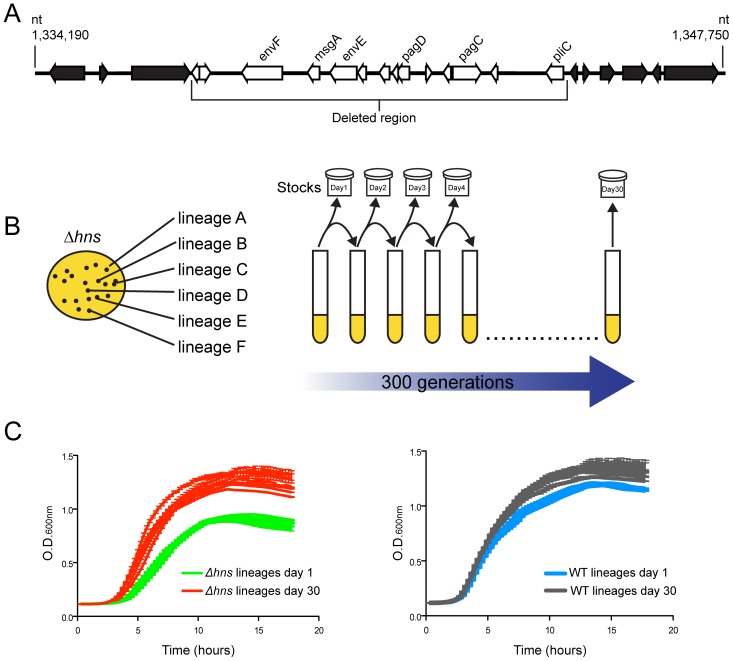
Experimental evolution of *S.* Typhimurium *hns* mutants. (A) An *hns* mutant isolate prepared for microarray analysis incurred a spontaneous 10 kb deletion after minimal laboratory passage. The white arrows represent the deleted open reading frames (ORFs) and the black arrows represent the ORFs adjacent to the deleted region. (B) Schematic diagram illustrating the experimental design of the serial passaging experiment. Six independently derived *S.* Typhimurium *hns* mutant colonies were selected to initiate cultures of LB media, named *Δhns* lineages A–F. The *Δhns* lineages were serially passaged in parallel with six wild type cultures for 30 days (approx. 300 generations) and samples from each lineage were stocked daily. Genomic DNA from each population at Day 1 and Day 30 of the evolution period was prepared for Illumina sequencing. (C) Left hand side: growth curves of the *hns* mutant populations at day 1 (green curves) and day 30 (red curves) of the evolution period. Right hand side: growth curves of the wild type populations at day 1 (blue curves) and day 30 (grey curves) of the evolution period. Liquid growth assays were performed in a 96-well plate reader.

In the course of an earlier microarray study of a *S.* Typhimurium Δhns/rpoS* strain we noted one isolate appeared to lose a large cluster of genes at some point during laboratory passage [22]. To identify the nature of this deletion the isolate was further analyzed by whole genome sequencing where reads were assembled against the *S.* Typhimurium 14028s reference genome (Genbank ID CP001363.1) using Geneious Pro 5.5.6 software. This analysis revealed that the isolate incurred a 10 kb genomic deletion spanning nucleotides 1,334,560 to 1,344,664 (Figure 1A). The deleted region is highly AT-rich (GC% = 40% as compared to the genome average of %GC = 52) and encodes several putative envelope proteins including the PhoP activated genes *pagC*, *pagD*, *pliC*, *envE*, *envF* and *msgA* [59]. Multiple studies have shown that expression of *pagC* is strongly repressed by H-NS, and the spontaneous loss of these genes from the *Δhns* isolate suggested that *hns* mutants are genetically unstable and may shed horizontally acquired sequences during passage [21], [22], [60].

We sought to experimentally determine if the loss of horizontally acquired sequences is a reproducible outcome of deleting *hns* from *S.* Typhimurium, as well as to identify novel compensatory mutations that may alleviate the fitness defects associated with the loss of H-NS. Toward this end an *in vitro* evolution screen was performed where six independently derived freshly constructed Δhns/rpoS* mutant lineages were serially passaged alongside six lineages of the isogenic rpoS* background (the “wild type” strain) in Luria-Bertani broth for 30 days, or approximately 300 generations (Figure 1B). The lineages were designated WT or *Δhns*, “A” through to “F”. Each day during the experiment, aliquots from the cultures were stocked and stored at −80°C to enable the retrospective analysis of genomic changes in each lineage over time. At the end of the evolution period, the growth rates of the passaged wild type and the passaged *Δhns* lineages were monitored alongside their unpassaged (day 0) counterparts (Figure 1C). All six lineages lacking H-NS displayed significant increases in their growth rates compared to their respective day 0 clone, while the wild type lineages displayed modest improvements in growth (Figure 1C). Notably, by day 30 the *Δhns* lineages all exhibited growth rates similar to that of the wild type strains at day 30.

### The *hns* mutant lineages evolved parallel genetic changes

**Figure 2:**
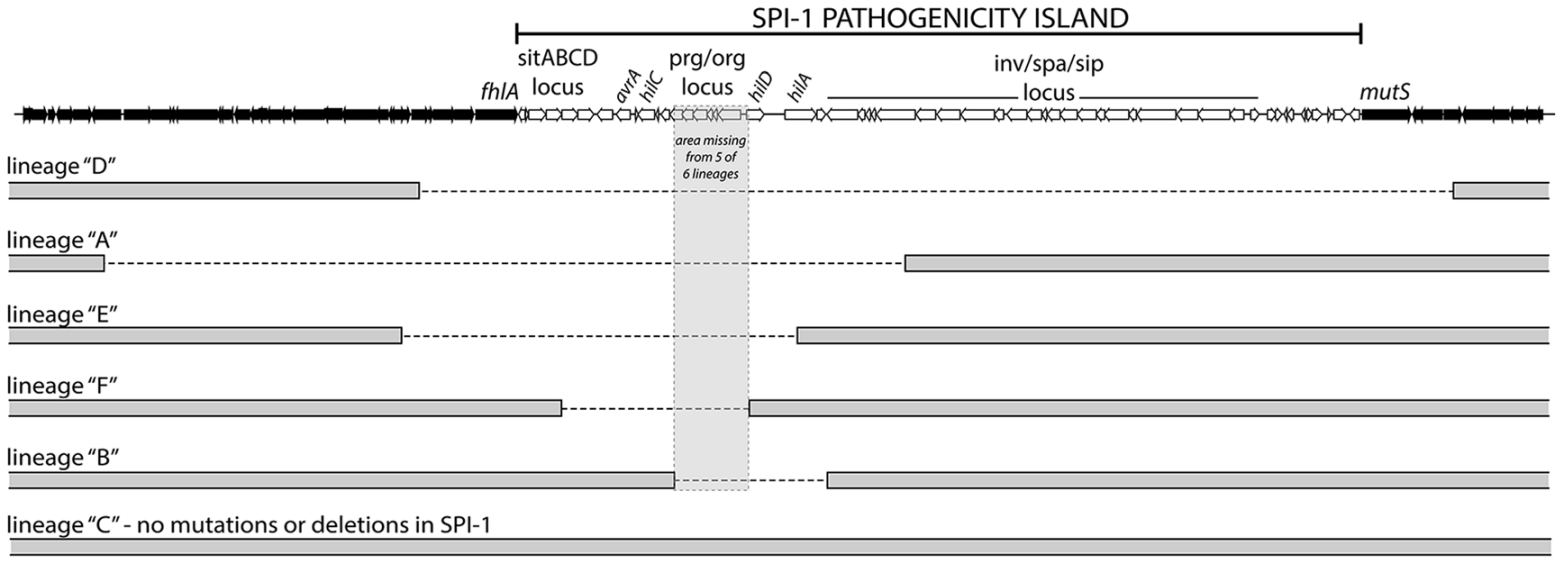
*S.* Typhimurium *hns* mutant lineages acquired large chromosomal deletions in the SPI-1 invasion locus. Throughout the 30-day evolution period, 5 out 6 *Δhns* lineages incurred 10–50 kb deletions in SPI-1. The lineages are arranged according to the size of the deletion they incurred. The deleted regions are marked with a dashed line. The grey box encompasses a region common to all five deletions that encoded the *prg/org* locus and the promoter and start codon of *hilD*. Lineage C did not acquire a deletion or any other mutations in the SPI-1 region.

To identify mutations that arose throughout the evolution period, genomic DNA from the passaged WT and *Δhns* lineages and their progenitor lines was analyzed by Illumina whole genome sequencing. In total, the six *Δhns* lineages acquired 15 missense mutations, 2 small deletions, 2 small insertions and 5 chromosomal deletions larger than 10 kb (Table 1). Most striking was the high degree of similarity in these mutations. Five of six *Δhns* lineages incurred unique 10–50 kb deletions within the *Salmonella* Pathogenicity Island 1 (SPI-1) and all six *Δhns* lineages accumulated missense mutations within the *stpA* gene encoding the H-NS paralogue StpA (Figure 2, Table 1).

**Table 1:** Summary of the genetic changes specific to the *hns* mutant lineages.

*Δhns* Lineage	Nucleotide Change	Amino Acid Change	Gene	Gene Product
A	A deletion	Frameshift	*phoP*	Transcriptional regulator
	C>T	T37I	*stpA*	H-NS paralog, DNA binding protein
	deletion		SPI-1	Spans nts. 3003900–3046122 (42 kb)
B	T>G	Y320D	*phoQ*	Sensor kinase
	C>T	T37I	*stpA*	H-NS paralogue
	GAA insert	E42 insert	*stpA*	H-NS paralogue
	deletion		SPI-1	Spans nts. 3033939–3042025 (8 kb)
C	G>A	G471D	*rpoD*	Primary RNAP sigma factor
	T>C	M4T	*stpA*	H-NS paralogue
	C>T	T37I	*stpA*	H-NS paralogue
D	T>C	V321A	*hyp*	Hypothetical protein
	C>T	P169S	*yecS*	Putative ABD-type AA permease
	G>A	D316N	*mutY*	Adenine DNA glycosylase
	A>G	M255V	*yhfC*	Hypothetical protein
	A>G	E62G	*idnK*	D-gluconate kinase
	GAA insert	E42 insert	*stpA*	H-NS paralogue
	C>T	T37I	*stpA*	H-NS paralogue
	deletion		SPI-1	Spans nts. 3029351–3074974 (55 kb)
E	A deletion	Frameshift	*phoQ*	Sensor kinase
	C>T	Silent	*stpA*	H-NS paralogue
	C>A	A77D	*stpA*	H-NS paralogue
	deletion		SPI-1	Spans nts. 3019555–3040455 (20 kb)
F	G>A	intergenic	-	nt. 3,432,191 in between genes *tdcA* and *rnpB*
	T>G	F76L	*stpA*	H-NS paralogue
	A>C	K38Q	*stpA*	H-NS paralogue
	deletion		SPI-1	Spans nts. 3027972–3038475 (10 kb)

In agreement with our earlier observations [22], three *Δhns* lineages acquired mutations in the genes encoding the PhoP/PhoQ two component system that activates many H-NS repressed genes involved in virulence, acid stress, resistance to antimicrobial peptides and intramacrophage survival [59], [61]–[64]. Specifically, lineages A and E acquired frameshift mutations in PhoP and PhoQ respectively while *Δhns* lineage B acquired a missense mutation (Y320D) in the cytoplasmic sensor kinase domain of PhoQ.

Throughout the experiment each *Δhns* lineage acquired a total of three to four mutations with the exception of *Δhns* lineage D, which acquired eight. It is notable that *Δhns* lineage D incurred the largest chromosomal deletion that extended beyond SPI-1 into the locus encoding *mutS* and *mutL*, essential components of the methyl-directed mismatch repair pathway [65]. The loss of either *mutS* or *mutL* from *E. coli* has been shown to result in a mutator phenotype and may explain the accumulation of other missense mutations specific to the *Δhns* lineage D, namely *idnK*(E62G), *mutY*(D316N), *yecS*(P169S), *yhfC*(M255V) and *stm1881*(V321A) [66]. Analysis of the SPI-1 deletion junction regions revealed that 3 of the 5 deletions occurred without any homology in the sequences flanking the deleted segment. The other 2 SPI-1 deletions occurred between segments homologous in only 4 nucleotides. This suggests that RecA mediated recombination did not play a role in the loss of this island in the *Δhns* mutants (Figure S1).

Analysis of the wild type lineages revealed that comparatively fewer genetic changes arose during the course of the experiment. 3 of the 6 wild type lineages acquired large chromosomal deletions that extended from 10 kb to 58 kb downstream of the *uvrC* locus (Table 2). Common to all three deleted fragments were components of the *uvrABC* nucleotide excision pathway and constituents of the flagellar apparatus. Under the laboratory growth conditions used in this study, expression of the *uvrABC* and flagellar genes likely resulted in a disadvantageous use of cellular resources. Apart from these deletions no mutations common among the wild type lineages were observed.

**Table 2:** Summary of the genetic changes specific to the wild-type lineages.

WT Lineage	Nucleotide Change	Gene	Gene Product
A	deletion	*uvrC-fliB*	Spans nts. 2048083–2058350 (10 kb)
C	deletion	*uvrC-fliG*	Spans nts. 2049159–2070524 (21 kb)
D	deletion	*uvrC-stm2443*	Spans nts. 2049052–2107464 (58 kb)
E	deletion	*stm3185*	Hypothetical protein, frameshift deletion spans nts. 3086531–3086545

### The temporal emergence of the mutations

To determine the timeline of the genetic changes that took place, genes of interest were PCR amplified from the frozen daily stocks of the *hns* mutant lineages and the PCR products were submitted for Sanger sequencing. This assay enabled the detection of mutant alleles soon after they arose in a given lineage and the relative proportion of the wild type and mutant alleles in the population at each day could be estimated from the sequencing chromatograms by analyzing the dual fluorescence peaks at a particular nucleotide. The relative signal strength of wild type vs. mutated nucleotides was used to approximate the emergence and dominance of each mutation in each population over time.

To determine when the large chromosomal SPI-1 deletions arose a PCR assay was employed; amplifying a region bridging the deleted segment. This detection method did not enable us to estimate the relative proportion of SPI-1 deletion strains in the population.

**Figure 3:**
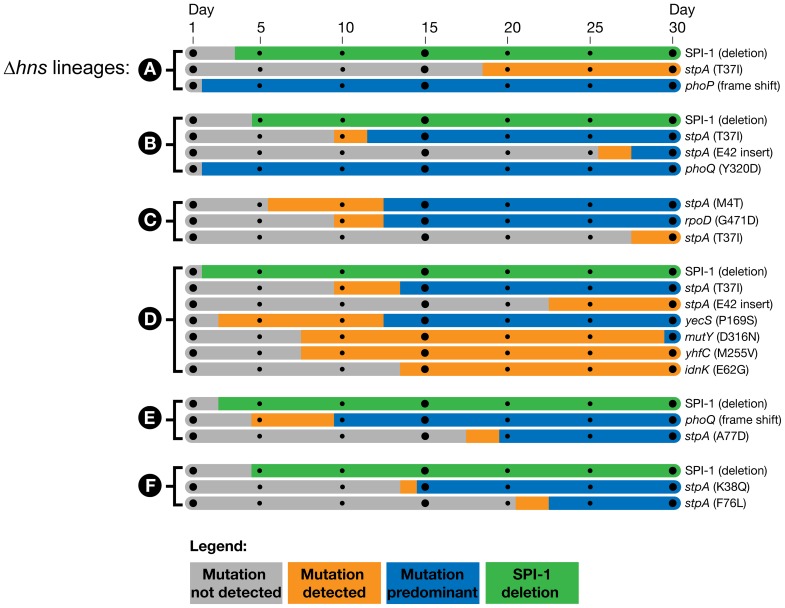
Timeline of the genetic changes in the *hns* mutant lineages. The mutations present in the *hns* mutant lineages at day 30 of the passaging period were detected by whole genome sequencing of the mixed populations. The identified genes of interest were PCR amplified directly from samples of the frozen daily culture stocks. The PCR products were sequenced and the relative signal intensities of the wild type vs. mutant alleles from each daily stock were used to estimate when the mutations emerged and became dominant in each population. Bars shaded in grey indicate the mutation was not detected via PCR sequencing, the orange bars indicate the mutation was detected in a fraction of the sequencing reads (<50%) and the blue bars indicate the mutant allele was predominant in the population (>50%). The large SPI-1 chromosomal deletions were detected by the appearance of a PCR product from the frozen stocks that spanned the deleted region. The green bars indicate detection of the SPI-1 deletions, the method employed did enable approximation of their abundance in the population.

We found the mutations in the PhoP/PhoQ regulatory system and the SPI-1 deletions were acquired by the *hns* mutant lineages in the early stages of the passaging period, prior to the missense mutations in *stpA* (Figure 3). The PhoP/PhoQ and SPI-1 mutations were detected as early as day 2 of the evolution period in *Δhns* lineages A, B and D, suggesting these mutations confer the greatest growth advantages and/or are most easily acquired.

Of particular interest is the *Δhns* lineage C, which did not obtain inactivating mutations in either the PhoP/PhoQ or SPI-1 but displayed a comparable increase in fitness as *Δhns* lineages A, B, D, E and F in liquid growth assays (Figure 1C). *Δhns* lineage C acquired a *stpA* missense mutation (M4T) by day 5 that persisted at low frequency until it also acquired a second mutation in the housekeeping sigma factor RpoD (G471D), at which point the *Δhns/stpA/rpoD* mutant rapidly outcompeted both the *Δhns* and *Δhns/stpA* mutant strains in the population by day 13. To address the concern that lineage C acquired SPI-1 inactivating mutations that were not detected with the Geneious Pro software we performed a reference alignment of the raw *Δhns* lineage C paired end reads to the *S.* Typhimurium 14028s reference genome using the Bowtie software package and also preformed a *de novo* genomic assembly of the evolved *Δhns* lineage C with Velvet [67], [68]. A list of variants from both the Bowtie and Velvet assemblies was generated with Samtools and no other mutations besides for the StpA_M4T_ and RpoD_G471D_ variants were identified [69].

### Inactivation of Salmonella Pathogenicity Island 1 improves growth of *hns* mutants

**Figure 4:**
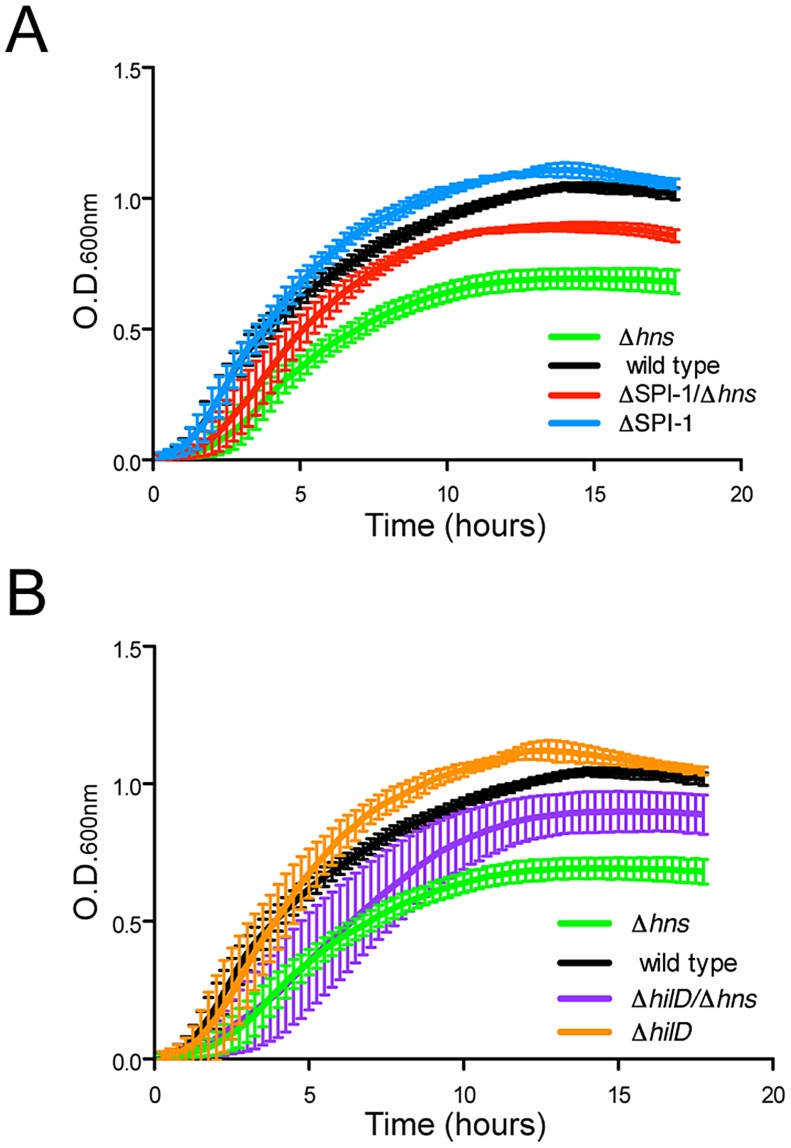
Disruption of SPI-1 expression improves fitness of an *hns* mutant. (A) Growth of a wild type *S.* Typhimurium (black curve) and a *Δhns* strain (green curve) was monitored in liquid media alongside wild type and *Δhns* strains harboring a 40 kb SPI-1 deletion (blue and red curves respectively). Elimination of the SPI-1 locus significantly increased growth of the *Δhns* strain and also provided a slight growth advantage in the wild type background. (B) Targeted disruption of *hilD*, the master SPI-1 activator produces a similar growth outcome as the SPI-1 deletion. Growth curves of the Δ*hilD* and *ΔhilD/Δhns* strains are represented in orange and purple respectively. The corresponding wild type (black) and *hns* mutant (green) growth curves are the same as panel A. Plotted is the average of three biological replicates and standard error (panels A and B).

The fact that five out of six *Δhns* lineages rapidly and independently incurred deletions within the SPI-1 locus suggested that SPI-1 misregulation is a major contributor to fitness defects in *S.* Typhimurium *Δhns* mutants. SPI-1 expression is repressed by *hns* and activated by a complex positive feedback loop where the production of the HilD regulatory protein induces the expression of HilA, a transcription factor that directly activates expression of the TTSS and effector proteins [70]. To determine the degree to which SPI-1 impairs growth of the *S.* Typhimurium *Δhns* mutant, we deleted the 40 kb genomic island from a wild type strain prior to introducing the *hns* deletion by transduction. The SPI-1 deletion significantly improved the growth of the *Δhn*s strain and also provided a mild improvement in growth of the wild type strain (Figure 4A). The region of SPI-1 lost in all *Δhns* lineages included the promoter upstream of *hilD*. Introduction of a *hilD* mutation into the *Δhns* background conferred a growth benefit similar to that of the 40 kb SPI-1 deletion (Figure 4B). These results indicate that in the absence of H-NS, SPI-1 is activated through a *hilD* dependent pathway and that the uncontrolled expression of SPI-1 encoded virulence determinants significantly impairs *Salmonella* growth.

*Salmonella enterica* harbors a second pathogenicity island, SPI-2, that encodes a type-3 secretion system distinct from the one encoded on SPI-1. Lucchini *et al.*, previously reported that construction of a *Salmonella ΔssrA*/*Δhn*s double mutant unable to express the genes encoded in SPI-2 significantly increased the growth rate of the *Δhns* strain (grown in LB media and using strain LT2) [21]. To determine if inactivation of SPI-2 encoded TTSS would offer the same fitness benefit as deletion of SPI-1 from a *Δhns* background, we introduced a 25 kb SPI-2 genomic deletion into *Δhns* and *Δhns/Δ*SPI-1 strains (Figure S2). Inactivation of SPI-2 did not significantly improve growth of either the *Δhns* or *Δhns/Δ*SPI-1 14028s strain to the same extent as loss of SPI-1. A similar experiment was conducted in LPM (low pH, low Mg^2+^ and low phosphate) media known to activate SPI-2 to determine if fitness of the *Δhn*s mutant would be adversely affected in a manner dependent on SPI-2. The *Δhn*s mutant failed to grow in this media but this growth defect was not alleviated in the *ΔssrA*/*Δhn*s double mutant indicating that other factors, not SPI-2, impact fitness in our strain under these particular conditions.

### Mutations in the H-NS paralog, StpA, provide partial complementation for the impaired growth and motility phenotypes of *hns* mutants

**Figure 5:**
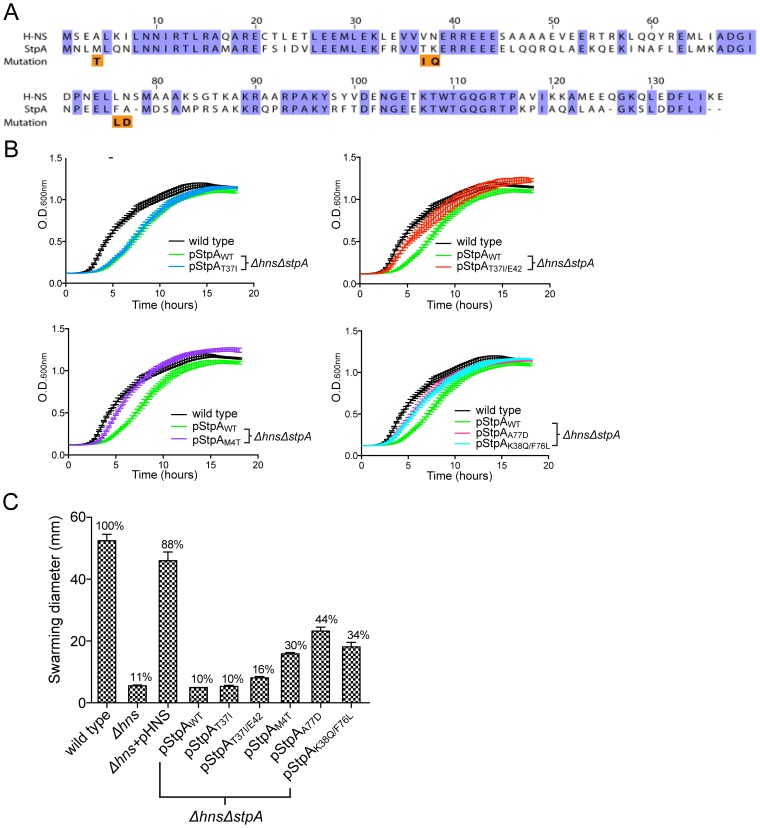
Missense mutations in the H-NS paralogue StpA partially restore the impaired growth and motility phenotypes of *hns* mutants. (A) Sequence alignment of H-NS and StpA from *S.* Typhimurium, conserved residues are highlighted in purple. The StpA amino acid substitutions acquired throughout the *hns* mutant passaging are indicated below the alignment in orange. (B) Plasmids harboring the StpA variants isolated from the passaged *Δhns* lineages were introduced into a *Δhns/ΔstpA* background and growth of the resulting strains was monitored in a 96-well plate reader. The wild type (black) and *Δhns/ΔstpA* strain expressing StpA_WT_ (green) growth curves are the same in each panel. (C) Swarming motility of the same strains used in the growth assays were measured on soft agar plates following a 12 hour incubation period at 37°C. Expression of StpA_T37I/E42_, StpA_M4T_, StpA_A77D_ and StpA_K38Q/F76L_ restored motility by 16%, 30%, 44% and 34% respectively. Bars represent the average swarm diameter of three independent experiments and error bars represent the standard error.

The only gene that acquired mutations in all six passaged *Δhns* lineages encodes the H-NS paralogue StpA. All of the acquired *stpA* mutations resulted in single amino acid substitutions or in-frame insertions that map to the predicted N-terminal and central dimerization domains of the protein. Because disruption of *stpA* in a *Δhns* background is known to exacerbate *hns* mutant phenotypes, we found it unlikely that these substitutions impaired *stpA* function. Intriguingly, the StpA mutations arise exclusively at sites where the unchanged amino acid is not conserved with H-NS, and the residue changes appear to render StpA more “H-NS-like” (Figure 5A). We hypothesized that the *stpA* mutations impart H-NS-like silencing properties to StpA and therefore partially compensated for the loss of *hns* at loci outside of SPI-1 in the serial passaging experiment.

To test the ability of the StpA variants to complement *hns* mutant phenotypes, we cloned the *stpA* locus from each passaged *Δhns* lineage and wild type *stpA* into a low copy vector with the native *stpA* promoter. The resulting plasmids were pStpA_WT_, pStpA_T37I_ cloned from *Δhns* lineage A, pStpA_T37I/E42ins_ from *Δhns* lineages B and D which both acquired the T37I substitution and an E42 insertion, pStpA_M4T_ from *Δhns* lineage C, pStpA_A77D_ from *Δhns* lineage E and pStpA_K38Q/F76L_ from *Δhns* lineage F. The StpA plasmids were transformed into a *Δhns/ΔstpA S.* Typhiumurim background in order to determine whether or not the isolated StpA variants could ameliorate bacterial fitness in the absence of *hns*. Introducing either pStpA_WT_ or StpA_T37I_ did not significantly improve growth of the *Δhns/ΔstpA* mutant (Figure 5B). On the other hand expression of the StpA_M4T_ mutant significantly improved bacterial fitness in the liquid growth assay. Likewise, the StpA_T37I/E42ins_ variant also offered an observable growth advantage. The strains expressing StpA_A77D_ and StpA_K38Q/F76L_ initially displayed a slight growth advantage and then plateaued at a similar final optical density as the StpA_WT_ expressing strain.

Given that expression of the StpA variants identified in the serial passaging experiment enhanced bacterial fitness to varying degrees, we next tested the ability of the modified StpA proteins to complement the impaired motility phenotype of *hns* mutants. H-NS is required for both the expression and assembly of a functional flagellum [71]–[73]. H-NS indirectly stimulates flagellar gene expression by repressing *hdfR*, a known repressor of the *flhDC* regulatory locus and, in addition, H-NS directly binds to the flagellar protein FliG and helps organize rotor subunit assembly [22], [74]. StpA has also been shown to bind FliG, but does not promote motility in the absence of H-NS unless cellular StpA levels are artificially elevated [74]. To determine if the StpA variants stimulate motility to a greater extent than wild type StpA, we employed the same strains used in the liquid growth assays and measured their radial swarming diameters on soft agar motility plates. After a 16 hr incubation period, wild type *S.* Typhimurium displayed a swarming diameter of 62 mm (Figure 5C). Similar to the *hns* mutant strain, the *S.* Typhimurium *Δhns/ΔstpA* strains harboring pStpA_WT_ and pStpA_T37I_ did not migrate beyond the original inoculation zone. Remarkably, the StpA variants StpA_M4T_, StpA_A77D_ and StpA_K38Q/F76L_ restored motility to the *Δhns/ΔstpA* strain by 30%, 44% and 34% that of the wild type strain respectively (Figure 5C). StpA_T37I/E42ins_ provided a small yet significant increase in swarming diameter to 16% the wild type diameter.

**Figure 6:**
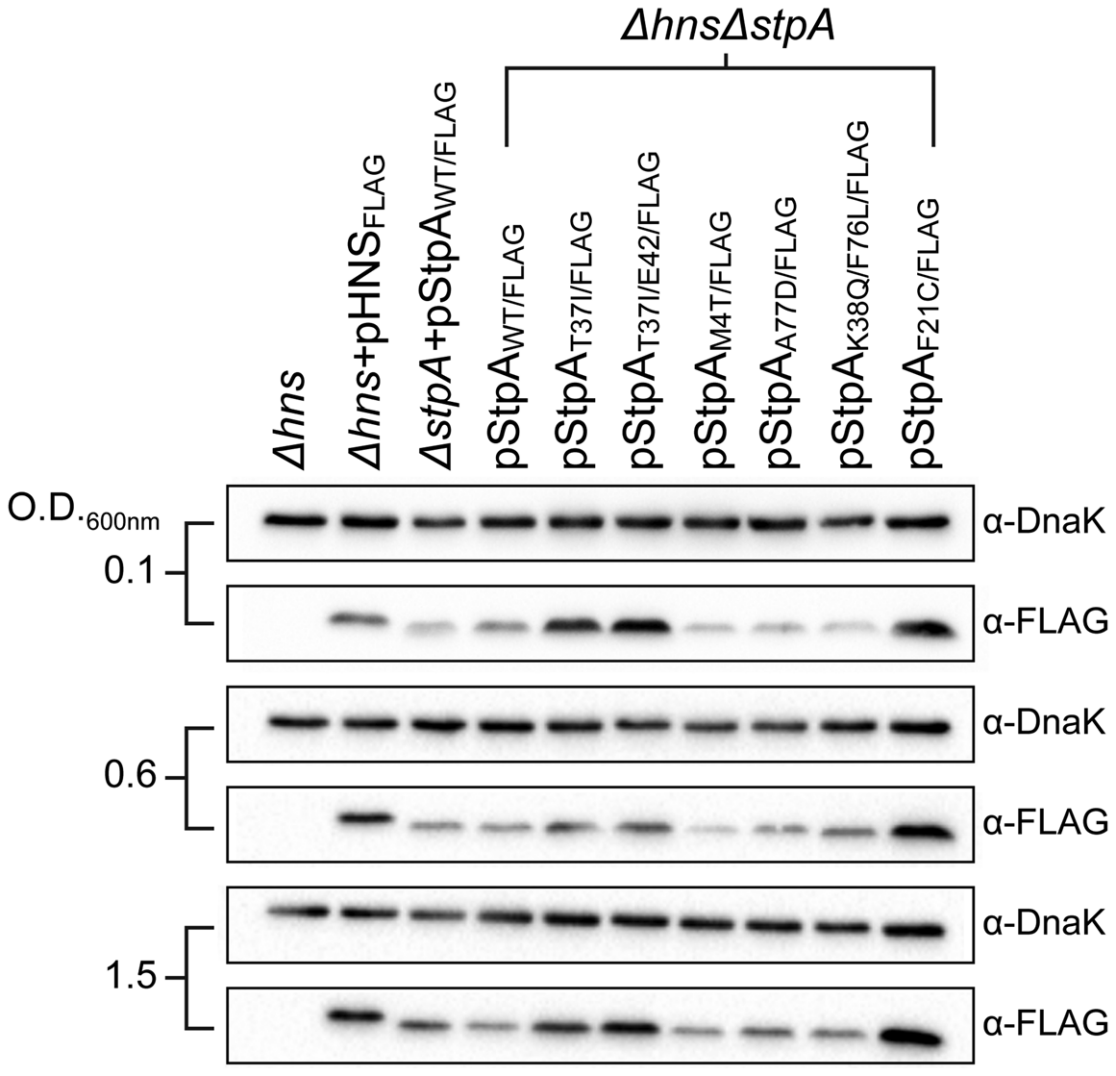
StpA missense mutations do not affect intracellular protein concentrations. Expression levels of FLAG-tagged StpA variants were monitored by western blot analysis at early exponential phase (O.D. 600 nm 0.1) mid exponential phase (O.D. 600 nm 0.6) and early stationary phase (O.D. 600 nm 1.5). α-DnaK served as a loading control.

One possibility by which the StpA variants could restore motility to the *Δhns* mutant would be if the single amino acid substitutions increase StpA protein stability. Intracellular StpA pools are reportedly subject to proteolysis by the Lon protease in strains lacking *hns* [56]. In this study a mutation in the N-terminal dimerization domain of StpA, F21C, was shown to impart resistance to proteolysis and increase intracellular StpA concentrations. To determine if any of the StpA mutations identified in our laboratory passage screen influenced protein levels, the amount of intracellular StpA was quantified by western blot analysis. *Δhns* strains harboring epitope tagged StpA or its variants was probed with an α-FLAG antibody. DnaK levels were analyzed on the same blot as a loading control. Similar to the StpA_F21C_ variant, StpA_T37I_ and StpA_T37I/E42_ accumulated to higher intracellular levels than StpA_WT_ (Figure 6)_._ In contrast the variants StpA_M4T_, StpA_A77D_ and StpA_K38Q/F76L_ were detected at similar levels to that of StpA_WT_. This suggests that the StpA variants identified in this study fall into one of two categories, mutations that increase intracellular StpA levels similar to the previously identified StpA_F21C_ variant, and a novel class of mutations that do not significantly alter intracellular StpA levels. Notably, it was the latter class of variants that provided partial complementation for the loss of *hns* in the growth and motility assays suggesting that the amino acid substitutions M4T, A77D and K38Q/F76L alter the functional properties of StpA and not its stability.

### Amino acid substitutions M4T and A77D alter StpA's silencing properties

Much like H-NS, StpA has also been implicated in silencing AT-rich regions of the genome. Although the set of genes under control of StpA shares significant overlap with the set of genes regulated by H-NS, in the absence of H-NS, the silencing activity of StpA alone does not provide sufficient repression of H-NS regulated loci [46], [48], [75]. To determine if the missense mutations acquired throughout the evolution of the *Δhns* lineages enhanced StpA's silencing activity, we measured the steady state transcript levels of four model H-NS and StpA regulated loci from a *Δhns/ΔstpA* strain harboring pStpA_WT_, pStpA_M4T_, pStpA_A77D_ and pStpA_F21C_. The StpA_M4T_ and StpA_A77D_ variants were chosen for transcript analysis because they provided the greatest restoration of the *Δhns* growth and motility defects without altering protein stability, while the StpA_F21C_ variant was included to determine the regulatory consequences of increased intracellular StpA levels. Also included in the analysis were a *Δhns* complemented strain (*Δhns*+pHNS) and a Δ*hns* strain, which served as reference points for repressed and derepressed transcript levels. cDNA from mid-log cultures was analyzed by Q-PCR with primers specific to *proV*, *hilA*, *ssrA* and *yciG*. *proV* is a well studied H-NS regulated gene target that resides outside the *Salmonella* pathogenicity islands, while *hilA* and *ssrA* are transcriptional activators encoded within SPI-1 and SPI-2 respectively. *yciG* is part of the *rpoS* regulon and was previously shown to be highly induced in a *Salmonella* SL1344 strain lacking *stpA* [48].

**Figure 7:**
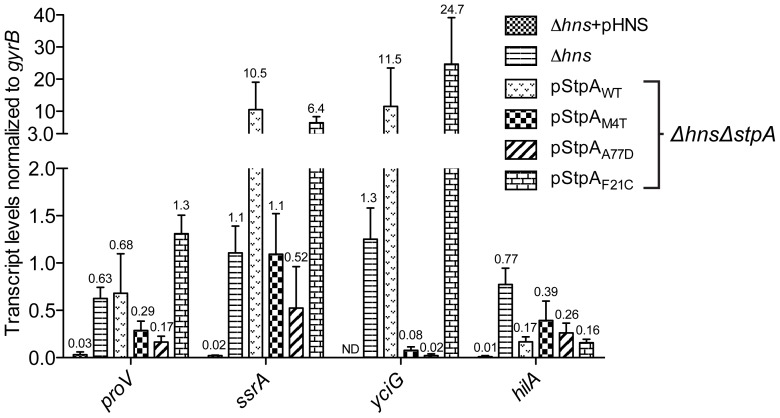
Point mutations A77D and M4T enhance StpA repression of select *hns* regulated loci. Transcript levels of four H-NS regulated genes, *proV*, *ssrA*, *yciG* and *hilA*, were measured by reverse transcriptase QPCR in a *Δhns/ΔstpA* strain expressing StpA_WT_ or the variants StpA_M4T_, StpA_A77D_ and StpA_F21C_. Transcript levels were also assayed in *Δhns* and *Δhns* complemented backgrounds (*Δhns*+pHNS).

Relative to the *Δhns* complemented strain, the transcript levels of *proV*, *hilA*, *ssrA* and *yciG* increased by 20-fold or greater in the *Δhns* strain (Figure 7). The expression of *yciG* is highly repressed in the *Δhns*+pHNS strain, its transcript levels were lower than the detection limit of the Q-PCR cycler and could not be reported with confidence. The *Δhns*/*ΔstpA* strain harboring pStpA_WT_ displayed a greater increase in the transcripts levels of *proV*, *ssrA* and *yciG* compared to the *Δhns* strain, while *hilA* transcript levels were reduced by 4.5-fold in the presence of pStpA_WT_. Substituting StpA_WT_ with StpA_M4T_ significantly reduced the expression levels of *proV* and *ssrA* by approximately 2-fold and 10-fold respectively. The StpA_A77D_ variant provided even greater repression of *proV* and *ssrA* by reducing their transcript levels by 4-fold and 20-fold relative to StpA_WT_. Similar to the *Δhns*+pHNS strain, both StpA_M4T_ and StpA_A77D_ maintained *yciG* expression levels close to the detection limit of the sensor. In contrast, the StpA_F21C_ variant that accumulates to higher intracellular levels than StpA_WT_ did not maintain significantly lower expression levels of any of the four genes tested relative to the pStpA_WT_ strain. This further establishes that the StpA_M4T_ and StpA_A77D_ variants as a novel set of mutations that enhance StpA silencing activity without affecting protein stability.

While the two single point mutations, M4T and A77D, significantly enhanced StpA's silencing activity at the *proV*, *ssrA* and *yciG* promoters regions these substitutions did not provide increased repression of *hilA*, encoding the SPI-1 transcriptional activator HilA. *hilA* expression is induced by three transcriptional activators, HilC, HilD and RtsA [70]. In the absence of H-NS it is possible that silencing complexes generated by StpA_M4T_ and StpA_A77D_, although more effective than StpA_WT_, were unable to impede the combined HilC and HilD-mediated activation of *hilA*.

### Mutations in *stpA* arise reproducibly as a consequence of mutations in *hns*

We repeated our *in vitro* evolution on an expanded number of freshly constructed *hns* deletion mutants to determine if loss of *hns* invariably led to mutations in *stpA* and, if so, to use this technique as a novel method of mapping functional residues in *stpA*. Toward this end *hns* deletion mutations were introduced by transduction into the *rpoS*-low strain to generate 12 independent lineages. To assess the impact SPI-1 may have on the evolution of *stpA* another 12 linages were generated by introducing the *hns* mutation into a strain already lacking SPI-1. Each of the 24 lineages were serially passaged in LB media over the course of 21 days and the *stpA* genes of each lineage were amplified by PCR and sequenced.

Sequencing of the *stpA* genes revealed missense mutations in 10/12 of the *hns* mutants in the *rpoS* background and 12/12 of the rpoS*/SPI-1 mutant background (Table 3). Remarkably the two *hns* mutant strains that did not acquire misssense mutations in *stpA* did acquire silent mutations, suggesting that either that *stpA* is prone to mutation in the absence of *hns* or that the presumably silent mutations actually affect StpA levels or function by increasing mRNA stability or by altering codon usage. As before all missense mutations mapped to the oligomerization domain between residues 2 and 80 of the *stpA* protein. Furthermore some lineages acquired as many as 4 different nucleotide substitutions. The fact that 30 independent lineages (24 in this experiment and 6 in the initial experiment) acquired mutations in *stpA* and that none of these were nonsense mutations confirms that there is strong selective pressure to acquire mutations in *stpA* in the absence of H-NS.

**Table 3:** Summary of *stpA* changes in second evolution experiment.

Lineage	Nucleotide Change	Amino Acid Change
A* (WT)	G>C 146, T>G 226	R49P, F76V
B* (WT)	T>A 111	Silent
C* (WT)	T>C 64, C>G 72	S22P, D24E
D* (WT)	A>C 114	K38N
E* (WT)	A>C 112, A>C 149	K38Q, Q50P
F* (WT)	A>C 67	I23L
G (WT)	A>C 114	K38E
H (WT)	A>C 112	K38Q
I (WT)	C>A 63, T>G 226	F21L, F76V
J (WT)	A>G 112	K38E
K (WT)	C>T 63	Silent
L (WT)	A>G 19, T>C 64, G>C 91	N7D, S22P, E31Q
M (Δ*SPI-1*)	A>G 19, C>T 110	N7D, T37I
N (Δ*SPI-1*)	T>C 61, T>A 228	F21L, F76L
O (Δ*SPI-1*)	A>G 19, T>C 61, C>T 110, G>C 146	N7D, F21L, T37I, R49P
P (Δ*SPI-1*)	A>G 19, C>T 110	N7D, T37I
Q (Δ*SPI-1*)	A>G 4, C>T 110, T>A 233	N2D, T37I, M78K
R (Δ*SPI-1*)	A>G 4, C>T 110	N2D, T37I
S (Δ*SPI-1*)	A>G 4, C>T 110, A>G 112	N2D, T37I
T (Δ*SPI-1*)	A>G 4, A>G 19, C>T 110	N2D, N7D, T37I
U (Δ*SPI-1*)	A>G 4, C>T110	N7D, T37I
V (Δ*SPI-1*)	A>G 4, C>T 110	N7D, T37I
W (Δ*SPI-1*)	A>G 67, C>T 110, T>G 226	I23V, T37I F76V
X (Δ*SPI-1*)	A>G 19, C>T 110, A>G 211	N7D, T37I, N71D

*Lineages A through F in this table are not the same as the A through F lineages derived from the first evolution experiment in Tables 3, 4 or Figures 1, 2, or 3.

Notably there were some differences observed in the specific mutations acquired between the two lineages (those with or without SPI-1). In the presence of SPI-1 the StpA protein was altered at several different residues but a cluster of mutations occurred at or near codon 38 (nucleotides 112–114) encoding lysine including a silent mutation at nucleotide 111. Strains that evolved in the absence of SPI-1 acquired a notably different set of mutations where all but one lineage acquired a mutation at nucleotide 110 resulting in the StpA(T37I) variant. Additional mutations changed the asparagine at positions 2 or 7 to an aspartic acid (N2D or N7D). This suggests that the pressures that select for mutations in StpA may differ in the absence of SPI-1.

**Figure 8:**
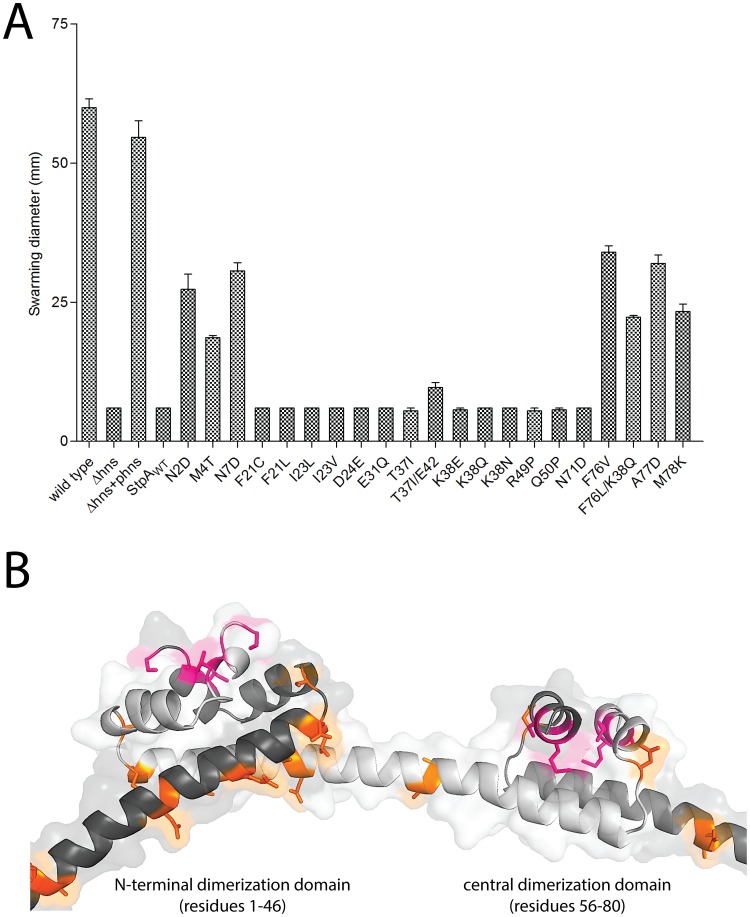
StpA mutants that partially restore the motility phenotype in *hns* mutants cluster in regions. (A) Swarming motility of the **s**trains expressing either StpA_WT_ or individual StpA variants were measured on soft agar plates following a 12 hour incubation period at 37°C. Bars represent the average swarm diameter of three independent experiments and error bars represent the standard error. (B) Location of the StpA mutations mapped on the structure of the H-NS oliomerization domain. Orange residues indicate individual changes that could not impart H-NS functionality upon StpA.

The results of the evolution experiment provided an opportunity to map what single or double residue changes in StpA would be sufficient to engender it with H-NS-like functionality. This functionality of each StpA variant was assessed by their ability to restore motility (Figure 8A) when expressed in the *hns* mutant background. This assay was chosen because our data with the earlier StpA variants indicated motility restoration correlates closely with their to silence H-NS regulated loci. These assays uncovered functional changes in single amino acids that cluster to two discrete regions of the StpA protein (Figure 8). The functional variants StpA_N2D_, StpA_M4T_, and StpA_N7D_ map to the short helix 1 that lies within the N-terminal dimerization domain while the variants StpA_F76V_, StpA_F76L_, StpA_A77D_ and StpA_M78K_ all map to helix 4 which is contained in the central dimerization domain. Other single residue StpA variants, where changes mapped to helix 3 or the short linker segments that connect helix 3 to the other helices, failed to restore significant motility to the *hns* mutant. Modeling these changes on the previously published H-NS oligomer structure show that the individual changes that confer H-NS-like function to StpA are buried within the dimerization interfaces or present on the outer, convex, surface of the H-NS filament while the residues that do not lie predominantly on the concave surface of the filament, and are largely predicted to have surface exposed side chains (Figure 8B). It is important to note the StpA residues were mostly assessed individually (only two double-mutants were assessed) and that some residues that appear to have no gain of function in our assays may have a more dramatic impact in combination with other changes.

### The M4T and A77D StpA variants, but not the T37I StpA variant, have DNA binding properties that are more similar to H-NS than to that of StpA

**Figure 9:**
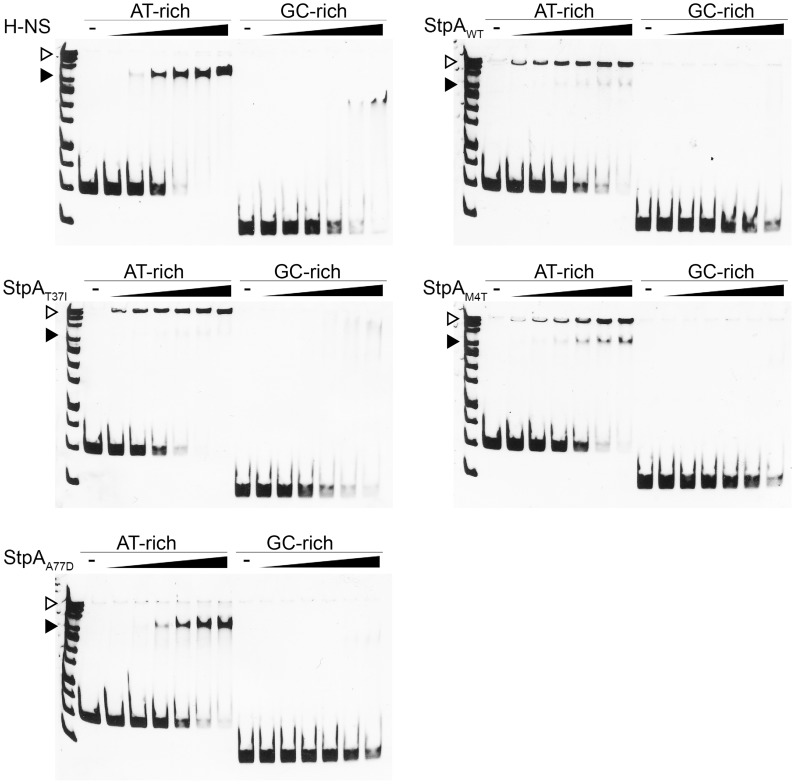
StpA_M4T_ and StpA_A77D_ form DNA-protein complexes more similar to H-NS than StpA_WT_. Electrophoretic mobility shift assays were conducted using purified H-NS, StpA_WT_, StpA_T37I_, StpA_M4T_, and StpA_A77D_. As indicated, the purified proteins were added to 289 bp AT-rich (%GC = 34) or 204 bp GC-rich (%GC = 74) model DNA fragments in increasing protein concentrations of 150 nM, 200 nM, 300 nM, 400 nM, and 500 nM. DNA/protein complexes were separated by 6% native polyacrylamide gel and stained with SYBR green. StpA complexes (▹) migrated alongside the top band of a nucleotide standard, while H-NS complexes (▸) migrated further into the gel as indicated.

Electrophoretic mobility shift assays were used to determine if changes in the StpA variants that led to increased “H-NS-like” function manifest as differences in their ability to form nucleoprotein complexes on DNA. Like H-NS, StpA_WT_ displays cooperative binding to a model 289 bp AT-rich sequence (%GC = 34) but forms nucleoprotein complexes are consistently observed to have significantly lower mobility than those formed by H-NS on the same DNA target (Figure 9). Remarkably the nucleoprotein complexes formed by the StpA_M4T_ and StpA_A77D_ variants formed complexes with motility more similar to H-NS than wild type StpA. StpA_M4T_ formed two complexes on DNA, one that migrated with the top band of the DNA ladder like StpA and one that migrated further into the gel at the same position as the H-NS complex. StpA_A77D_ almost exclusively formed a single H-NS like complex. StpA_T37I,_ which had enhanced protein levels *in vivo*, but failed to complement for H-NS for either motility or silencing, formed a lower mobility nucleoprotein complex identical to that of the wild type StpA protein. Notably, there were no differences in overall affinity for DNA between the different variants.

**Figure 10:**
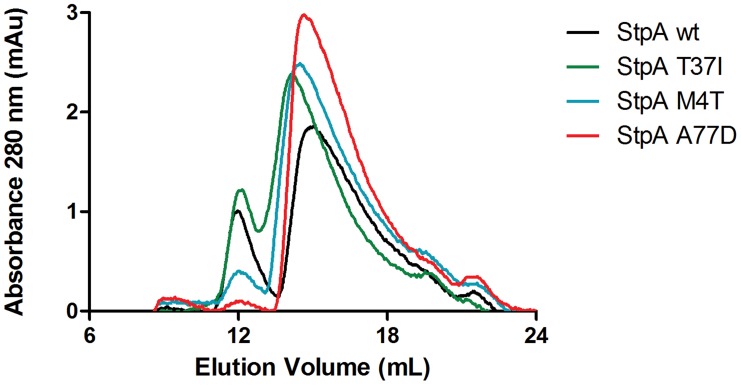
Analytical gel filtration of StpA and the StpA_M4T_, StpA_A77D_, and StpA_T37I_ variants. Purified StpA_WT_, StpA_T37I_, StpA_M4T_, StpA_A77D_ proteins at a concentration of 100 µM were applied to a Superdex 200 10/300 GL column pre-equilibrated with 20 mM Tris pH 8, 1 M NaCl, 1 mM EDTA and 5% glycerol. The column was calibrated using globular protein standards ranging from 12.4 kDa to 443 kDa.

This data indicates that subtle changes in the dimerization domains of StpA can generate large and quantifiable differences in properties of the nucleoprotein complex and that the functional differences observed between StpA variants manifest as differences in their effects on nucleoprotein structure. At high protein concentrations both StpA and H-NS have the ability to spontaneously oligomerize into higher order structures in the absence of DNA, a phenomenon that can be measured by changes by analytical gel filtration chromatography. We assessed the gel filtration profiles of StpA and its variants (Figure 10) to determine if any changes in their oligomerization states could be observed. StpA_WT_ and the StpA_T37I_, which do not effectively substitute for H-NS, displayed two prominent peaks with calculated molecular weights of approximately 450 and 150 kDa (StpA monomer is ∼15 kDa). The chromatographic profiles of the StpA_M4T_ and StpA_A77D_ proteins indicate that these proteins have a dramatically reduced propensity to form the oligomeric species that elutes early during chromatography. We note that the asymmetrical rod-like structure of the StpA and H-NS oligomers prevent an accurate determination of molecular weight based on mobility through the column when compared to a set of globular standards. Differences in shape or flexibility would also manifest as different elution profiles by gel filtration. Nevertheless these findings when taken as a whole indicate that the functional differences between the StpA variants (and also the functional differences between H-NS and StpA) are primarily due to differences in manner of their oligomerization and not in the specificity of their DNA binding domains.

## Discussion

The xenogeneic silencing model predicts that the selective silencing of foreign DNA accelerates bacterial evolution by reducing the fitness cost associated with HGT. While multiple studies have established a role for the H-NS, MvaT and Lsr2 protein families in regulating newly acquired sequences, the evolutionary advantage of foreign gene repression by H-NS and its impact on genome content had not been assessed by experimental evolution [21], [22], [39], [40]. The genetic adaptations we identify that improve growth in strains lacking H-NS indicate that xenogeneic silencing played a major role in the evolution of the Salmonellae by buffering the fitness consequences caused by the SPI-1 encoded TTSS, a defining characteristic of the species. Indeed a recent study on the evolution of *Salmonella* revealed that, while many sequences acquired by HGT will adopt the %GC of their host over time, the major pathogenicity islands have selectively retained their AT-richness, presumably to maintain their silencing by H-NS [6]. The fact that we observed large deletions in SPI-1, rather than inactivating point mutations or small indels, is somewhat surprising and suggests that this region may be naturally unstable and prone to gene loss. The 5 deletions independently occurred at different sequences, each with limited no flanking homology, suggesting that replication errors and not homologous or site-specific recombination likely caused the loss of these regions from the genome.

Multiple lines of evidence suggest that maintaining a TTSS represents a costly investment of cellular resources. Induction of the *Yersinia* TTSS by low calcium essentially halts bacterial growth and the plasmid-encoded *Shigella* TTSS is readily lost during laboratory passage [20]. An association between impaired bacterial growth and SPI-1 expression in wild type cells was recently reported in a study conducted by Sturm *et al* [76]. This study tracked the spontaneous induction of the SPI-1 encoded TTSS at a single cell level using time-lapse microscopy imaging. Sturm *et al.* correlated the expression of the TTSS with retarded growth rates that were alleviated by mutations in the SPI-1 activator *hilA*. Under the conditions employed in this study the *hns* mutant strains only incurred genomic deletions within SPI-1. However, a targeted disruption of SPI-2 was previously shown to partly improve growth in an *hns* mutant background [21]. We believe the loss of SPI-1 and not SPI-2 from our *hns* mutant lineages likely reflects that the conditions we employed in this study favored SPI-1 expression. It is important to note that the conditions employed during the experimental evolution experiment were arbitrarily chosen and it is entirely likely that subtle changes in environment will significantly impact which loci will impinge on fitness in the absence of H-NS. While the wild type lineages passaged in parallel to our *hns* mutants did not acquire mutations in the SPI-1 locus, genomic deletions encompassing components of the flagellar apparatus were noted in 3 out of 6 of the wild type lineages. Flagella and TTSS are evolutionarily related and highly homologous in both primary sequence and structure [77]. The fact that the flagellar loci of the *hns* mutant lineages did not acquire mutations is consistent with the fact that *hns* mutants fail to express flagella to begin with.

Pathogens like *Salmonella* spend a substantial amount of time outside of the host environment and our studies suggest that H-NS is essential for enteric bacteria to retain virulence in the absence of selective pressures. Naturally occurring SPI-I deletions have occasionally been identified among environmental *Salmonella* isolates that have consequentially lost the ability to invade host cells [78]. Spontaneous SPI-1 mutations are thought to arise throughout host infection generating a subpopulation of “avirulent defectors” that propagate much faster than their TTSS-positive predecessors [79]. Diard *et al* demonstrated that *Salmonella* infection with a constitutively active SPI-1 TTSS strain resulted in a sharp rise of the genetically avirulent subpopulation and consequently premature clearing of the infection [79].

The only *hns* mutant lineage that did not incur a SPI-1 deletion, *Δhns* lineage C, acquired a missense mutation in *rpoD* (RpoD_G471D_). The recent crystal structure of the *E. coli* RNAP/σ^D^ holoenzyme shows RpoD residue G471 is located in an exposed loop region, enriched in highly conserved aromatic and positively charged residues [80]. An alignment of the *E. coli* RNAP/σ^D^ structure with the RNAP/σ^D^ initiation complex from *Thermus thermophilus* reveals the conserved loop region harboring residue G471 is in close proximity the template strand during transcriptional initiation [81]. It is possible that introduction of the negatively charged aspartic acid residue at position 471 could hinder transcriptional initiation and result in reduced expression of the SPI-1 locus, however it is currently unclear how this mutation would affect SPI-1 but not impede the expression of many other important σ^D^ targets.

Another central and important outcome of this study was the identification StpA oligomerization variants that partially compensate for several H-NS dependent phenotypes. Many of these mutations do not increase cellular StpA protein concentrations, as has been observed previously [46], [54]. Notably a recent study on a spontaneous mutant that improved fitness of an *E. coli* strain lacking Hha and YdgT, two molecules that collaborate with H-NS to facilitate gene silencing, identified a promoter mutation that dramatically enhanced H-NS levels [82]. StpA in *S.* Typhimurium was recently proposed to repress the *rpoS* regulon during exponential growth and the major caveat of our study is that we started with strain encoding a defective RpoS, which would alleviate the selective pressure to maintain a wild type copy of StpA [48]. H-NS also represses numerous genes activated by *rpoS* in response to cellular stress [83]–[85]. A study of the *hdeAB* promoter region suggested H-NS repression was overcome by the RNAP•σ^S^ complex, while RNAP associated with the house keeping sigma factor σ^D^ was more effectively inhibited by the presence of H-NS [85]. A similar finding was also reported for the *dps* promoter [86]. One model that could be extrapolated from these observations is that StpA restricts transcription of the RNAP complexed with σ^S^ while H-NS more efficiently represses RNAP bound to σ^D^. Complicating this model is the fact that H-NS and StpA can heterodimerize and that each may individually regulate cellular σ^S^ concentrations [48]–[50], [87], [88].

The story that is emerging from this and other recent studies is that subtle changes in local nucleoid architecture, directed by the structure of the oligomerized protein, underlies the diverse functions ascribed to the H-NS like molecules. Several findings indicate that changes in DNA shape and tension are the relevant outputs of this class of transcriptional modulators; a mode of gene regulation that is particularly challenging to study using conventional assays like EMSA and footprinting. Our results indicate that StpA and H-NS differ primarily not in their ability to bind AT-rich DNA *per se*, in fact StpA binds DNA with an apparent affinity higher than that of H-NS, but by some physical property that manifests as a change in promoter architecture once bound by the protein. Due to its apparent higher affinity for DNA and elevated propensity to form higher-order oligomers in conventional assays one would predict that StpA would be a more effective silencer than H-NS in most situations. We note that there are significant qualitative differences in the shifted DNA complexes between the StpA variants that can complement for the loss of H-NS and those variants that cannot. This supposition is further supported by recent studies on the H-NS-like transcriptional activator Ler and H-NS paralogs encoded on plasmids demonstrating that the central linking domain, not the DNA binding domain, is the primary determinant in how these molecules functionally differ from H-NS [89], [90].

Evidence that H-NS, StpA, and the “H-NS-like” Ler proteins each form characteristically distinct higher order protein/DNA complexes has been more directly provided by recent atomic force microscopy imaging studies and single molecule “DNA stretching” experiments [91]–[94]. Lim et al reported that StpA-induced DNA/protein filaments were significantly more rigid than those produced by H-NS, and that the StpA filaments were insensitive to changes in pH, temperature, and osmolarity; conditions known to disrupt H-NS-DNA binding [92]. Another observation that might support divergent oligomerization properties of StpA and H-NS is that StpA can silence the *E.coli bglG* operon, but only in the presence of H-NS molecules deficient in DNA binding [50], [95]. This observation was used to suggest that the H-NS proteins can heterodimerize with StpA to facilitate silencing of *bglG*. However, based on our new findings, we cannot exclude the possibility that the *hns* mutant strain used in that study acquired mutation(s) in *stpA* during routine lab passaging that enabled it to act like H-NS.

The fact that compensatory *stpA* mutations arise rapidly and reliably in the absence of H-NS is a worrying outcome of this study. Complicating matters further is the apparent functional heterogeneity in the various *stpA* mutations we uncovered, i.e. the different compensatory mutations do not share the exact same properties. It is unclear how much care has been taken in the maintenance of the various *hns* mutant strains employed in many prior studies and in all but one case it is clear that the *stpA* locus was not sequenced to check for mutations. Regrettably this leaves some doubt regarding the validity of earlier studies on the phenotypes of strains lacking H-NS. Given their genetic instability, all future work on *hns* mutants in either *E. coli* or *Salmonella* should be performed on multiple freshly constructed (transduced) isolates and laboratory passaging of such strains should be kept to a minimum. Whenever possible the genomes of *hns* mutants should be re-sequenced to verify that phenotypes ascribed to H-NS are not, in fact, due to a mutation in a different gene.

## Materials and Methods

### Plasmid and strain construction

The plasmids and strains employed in this study are listed in Table 4 and a complete list of oligonucleotides sequences is provided in Table 5. In a previous study, a FLAG-epitope tag was incorporated into the XhoI and BamHI sites of the low copy vector pHSG576 to generate pWN425 [22]. The *stpA* coding sequence and 206 bp upstream region (comprising nucleotides 2976460 to 2968067 in the *S.* Typhimurium 14028s genome Genbank ID CP001363.1) was PCR-amplified from *S.* Typhimurium 14028s genomic DNA with primers ALO115 and ALO116. The amplified fragment was ligated into the PstI and BamHI sites of pHSG576 backbone for expression of StpA harboring a C-terminal FLAG epitope tag. The StpA coding sequence and promoter region were incorporated into pHSG576 in the opposite orientation of the lac promoter, such that *stpA* expression levels were controlled by the native *stpA* promoter. The resulting plasmid pStpA_WT_ was used for complementation studies. Similarly, plasmids harboring the StpA variants identified in the experimental evolution screen were constructed using the pHSG576 backbone with a C-terminal FLAG epitope tag. The mutated StpA alleles were PCR amplified from the genomic DNA of their respective *hns* mutant lineages that had been passaged for 30 days. The same primer pair used to amplify the wild type *stpA* coding sequence and 5′ promoter region was employed. The mutant *stpA* allele PCR fragments were inserted into the PstI and BamHI sites of vector pHSG576 harboring the FLAG epitope sequence 3′ of the BamHI site. The plasmids generated and the corresponding *hns* mutant lineage that the *stpA* alleles were cloned from were as follows: pStpA_T37I_ from *Δhns* lineage A, pStpA_T37I/E42insert_ from *Δhns* lineages B and D, pStpA_M4T_ from *Δhns* lineage C, pStpA_A77D_ from *Δhns* lineage E and pStpA_K38Q/F76L_ from *Δhns* lineage F. The sequences of all the plasmids constructed in this study were confirmed by Sanger Sequencing at the TCAG Sequencing Facility (Centre for Applied Genomics, Hospital for Sick Children).

**Table 4:** Plasmids and strains used in this study.

Plasmid or strain	Description		Reference
Plasmids		Vector	
pStpA_WT_	Low copy vector encoding StpA_WT_ with C-terminal FLAG epitope, under control of the *stpA* promoter. Variants listed below are constructed identically.	pHSG576	This study
pStpA_T37I_	FLAG-tagged StpA_T37I_	pHSG576	This study
pStpA_T37I/E42_	FLAG-tagged StpA_T37I/E42_	pHSG576	This study
pStpA_M4T_	FLAG-tagged StpA_M4T_	pHSG576	This study
pStpA_A77D_	FLAG-tagged StpA_A77D_	pHSG576	This study
pStpA_K38Q/F76L_	FLAG-tagged StpA_K38Q/F26L_	pHSG576	This study
pStpA_N2D_	FLAG-tagged pStpA_N2D_	pHSG576	This study
pStpA_N7D_	FLAG-tagged pStpA_N7D_	pHSG576	This study
pStpA_F21C_	FLAG-tagged pStpA_F21C_	pHSG576	This study
pStpA_F21L_	FLAG-tagged pStpA_F21L_	pHSG576	This study
pStpA_S22P_	FLAG-tagged pStpA_S22P_	pHSG576	This study
pStpA_I23L_	FLAG-tagged pStpA_I23L_	pHSG576	This study
pStpA_I23V_	FLAG-tagged pStpA_I23V_	pHSG576	This study
pStpA_D24E_	FLAG-tagged pStpA_D24E_	pHSG576	This study
pStpA_E31Q_	FLAG-tagged pStpA_E31Q_	pHSG576	This study
pStpA_K38N_	FLAG-tagged pStpA_K38N_	pHSG576	This study
pStpA_K38Q_	FLAG-tagged pStpA_K38Q_	pHSG576	This study
pStpA_K38E_	FLAG-tagged pStpA_K38E_	pHSG576	This study
pStpA**_R_**_49P_	FLAG-tagged pStpA_R49P_	pHSG576	This study
pStpA_Q50P_	FLAG-tagged pStpA_Q50P_	pHSG576	This study
pStpA_N71D_	FLAG-tagged pStpA_N71D_	pHSG576	This study
pStpA_F76V_	FLAG-tagged pStpA_F76V_	pHSG576	This study
pStpA_M78K_	FLAG-tagged pStpA_M78K_	pHSG576	This study
pWN425 (pHNS_WT_)	FLAG-tagged H-NS	pHSG576	[22]
pET21b StpA**_wt_**	Inducible expression of His-tag StpA_wt_		This study
pET21b StpA_T37I_	Inducible expression of His-tag StpA_T37I_		This study
pET21b StpA_M4T_	Inducible expression of His-tag StpA_M4T_		This study
pET21b StpA_A77D_	Inducible expression of His-tag StpA_A77D_		This study
Strains			
WN153	*S.enterica* serovar Typhimurium 14028s rpoS* (five amino acid in frame deletion that reduces RpoS activity)		[34]
SSA105	WN153 Δ*hns*::Km/pHSG576 vector only		[22]
SSA162	WN153 Δ*stpA*Δ*hns*::Km/pStpA_WT_		This study
SSA163	WN153 Δ*stpA*Δ*hns*::Km/pStpA_T37I_		This study
SSA164	WN153 Δ*stpA*Δ*hns*::Km/pStpA_T37I/E42insert_		This study
SSA165	WN153 Δ*stpA*Δ*hns*::Km/pStpA_M4T_		This study
SSA166	WN153 Δ*stpA*Δ*hns*::Km/pStpA_A77D_		This study
SSA167	WN153 Δ*stpA*Δ*hns*::Km/pStpA_K38Q/F76L_		This study
SSA171	WN153 ΔSPI-2::CmΔ*hns*::Km		This study
SSA172	WN153 ΔSPI-1 ΔSPI-2::CmΔ*hns*::Km		This study
JS008	BL21 Δ*hns*/pET21b StpA_wt_		This study
JS009	BL21 Δ*hns*/pET21b StpA_T37I_		This study
JS011	BL21 Δ*hns*/pET21b StpA_M4T_		This study
JS012	BL21 Δ*hns*/pET21b StpA_A77D_		This study
JS065	WN153 Δ*stpA*Δ*hns*::Km/pStpA_F21C_		This study
JS076	WN153 Δ*stpA*Δ*hns*::Km/pStpA_N7D_		This study
JS077	WN153 Δ*stpA*Δ*hns*::Km/pStpA_F21L_		This study
JS078	WN153 Δ*stpA*Δ*hns*::Km/pStpA_I23L_		This study
JS079	WN153 Δ*stpA*Δ*hns*::Km/pStpA_D24E_		This study
JS080	WN153 Δ*stpA*Δ*hns*::Km/pStpA_K38E_		This study
JS081	WN153 Δ*stpA*Δ*hns*::Km/pStpAF_76V_		This study
JS082	WN153 Δ*stpA*Δ*hns*::Km/pStpA_M78K_		This study
JS083	WN153 Δ*stpA*Δ*hns*::Km/pStpA_I23V_		This study
JS084	WN153 Δ*stpA*Δ*hns*::Km/pStpA_R49P_		This study
JS085	WN153 Δ*stpA*Δ*hns*::Km/pStpA_Q50P_		This study
JS086	WN153 Δ*stpA*Δ*hns*::Km/pStpA_S22P_		This study
JS087	WN153 Δ*stpA*Δ*hns*::Km/pStpA_K38Q_		This study
JS088	WN153 Δ*stpA*Δ*hns*::Km/pStpA_K38N_		This study
JS089	WN153 Δ*stpA*Δ*hns*::Km/pStpA_N2D_		This study
JS090	WN153 Δ*stpA*Δ*hns*::Km/pStpA_E31Q_		This study
JS091	WN153 Δ*stpA*Δ*hns*::Km/pStpA_N71D_		This study
AL089	WN153 ΔSPI-1::Cm		This study
AL108	WN153 Δ*hilD*		Laboratory of Dr. Fang
AL109	WN153 ΔSPI-1::Cm Δ*hns*::Km		This study
AL113	WN153 ΔSPI-2::Cm		This study
AL118	WN153 Δ*hilD*Δ*hns*::Km		This study

**Table 5:** Oligonucleotides used in this study.

Name	Sequence
ALO76	5′TATGAAGCGATTGGGTATTGATAAAGACGCGTTAGCGTAAGTGTAGGCTGGAGCTGCTTC 3′
ALO77	5′TACATTCTCATCATTCCTGGACTACAACAGGGGTGATACTCATATGAATATCCTCCTTAG 3′
ALO83	5′TTAACCTTCGCAGTGGCCTGAAGAAGCATACCAAAAGCATCATATGAATATCCTCCTTAG 3′
ALO84	5′CAAAATATGACCAATGCTTAATACCATCGGACGCCCCTGGGTGTAGGCTGGAGCTGCTTC 3′
ALO115	5′AAAACTGCAGTTAGATTAAGAAATCATCCAGAG 3′
ALO116	5′AAAAGGATCCAAACCTACAGATATACCGTAG 3′
ALO117	5′ATAGAGACAGGAAACGAAGCGCCA 3′
ALO118	5′CAATCGCATACAATACCGCCTGTAATAG 3′
ALO122	5′TCACGGTTTAAGCAGCGAAGAACG 3′
ALO123	5′AAATTCTGCAGGCCAAATAGGGCG 3′
ALO127	5′GTTGGCGACGTCGGTTTCCAG 3′
ALO128	5′ GCAGCAATCTTACGCACCTTGTTGAC 3′
ALO129	5′ GGTGCCCGGTTCAACTTTTGC 3′
ALO130	5′ GTTTGCCGCGATAGAAAGTCTTTTGT 3′
ALO131	5′ GGAAGCCGAATGCTGGTGTGAG 3′
ALO132	5′ GGAGATCGGGATGGCGCTG 3′
ALO133	5′ GCAACGCCACATCGCGAATC 3′
ALO134	5′ CAGGTGATAACCTTTAACCCGAACTATCTC 3′
ALO135	5′ GCAACAGTCAGTCAACAAGGCAC 3′
ALO136	5′ CTTCCACTGTCTACTGGGTGACGAAG 3′
ALO139	5′CGTAATATTGTGTTGTGGAATATGACTCAGAGG 3′
ALO140	5′GACTTCTGACCGCCTGACTCAGC 3′
ALO141	5′GTGTTTGAGTTCTGAAAACGGGCATTATCC 3′
ALO142	5′GTTCTGCTCATAAGTCATCCTCTTCATCG 3′
ALO143	5′GGTATTCCATGTTAAGTATCCGCAGGCTG 3′
ALO144	5′CCAGTATCCGCACGATGTCATTACCAC 3′
ALO145	5′CCAGCGCCACTATGCCATATGC 3′
ALO146	5′GCACCATAATCAACGCTAGACTGTTC 3′
ALO151	5′CGTGCAGCACCGTTTGACCATG 3′
ALO152	5′CTGCGCTGTTTGCCTACCATCC 3′
ALO153	5′CCTCCACGCGCTTCACTTCTCTTC 3′
GT49	5′CCGCAGGTGGCTGAACA
GT50	5′CGAATGCGGTGCGTTGATGG
GT68	5′GGCATGATAATAGTGTATTCTCTT
GT77	5′CTCTCTCTGCACCAGGATA
SSA198	5′TCGGGAAGTTTAACCGTCACCTCA 3′
SSA199	5′ACATAAACGGCAAGGGATGGCACG 3′
SSA200	5′ATATGCCGTTCTGGTCATCCTGCT 3′
SSA201	5′TCCGACAGAATACGTCGTAAGGCA 3′
SSA202	5′TCTGGCCATTGAAGAAGGCGAGAT 3′
SSA203	5′TCAACGCCGTCAATCAGTACCTGT 3′
SSA232	5′CATCGTGGTGGTTCAGGAAAT 3′
SSA233	5′TGAAATTCCCGCCGCTATG 3′
WNp233	5′GATGGGTTTTCCAGCAGGTATTC 3′
WNp234	5′AGGTCTGATTGCGGTGGTTTC 3′

The *Salmonella enterica* serovar Typhimurium 14028s strains used in this study possess a mutant *rpoS* allele (called rpoS*) that encodes a five residue in-frame deletion that significantly reduces RpoS (σ^32^) activity [22]. The single *stpA* and *hns* chromosomal deletion strains were previously constructed using the lambda red recombinase method described by Datsenko and Wanner [96]. The *stpA* gene from *S.* Typhimurium 14028s was replaced with a kanamycin resistance cassette amplified from plasmid pKD4, flanked by FRT recombinase sites. The kanamycin resistance cassette was subsequently flipped out of the chromosome by introducing the pCP20 plasmid expressing the FLP recombinase. This generated a *S.* Typhimurium Δ*stpA* strain without antibiotic resistance markers. To test the ability of the StpA variants to compensate for the loss of H-NS, each of the StpA complementation plasmids were transformed into the *S.* Typhimurium 14028s *ΔstpA* mutant. Next, the *hns* null allele was moved into the Δ*stpA* mutant strains harboring the StpA complementation plasmids by P22 transduction. The resulting clones were selected for on Miller's Luria Bertani (LB) 1% agar plates supplemented with 50 µg/ml kanamycin (to select for the *hns* null mutation) and 20 µg/ml chloramphenicol (to select to the StpA plasmids).

SPI-1 deletion mutants were constructed by deleting a 44.4 kb region spanning the SPI-1 region using the lambda red recombinase method described by Datsenko and Wanner [96]. In brief, the region between *S.* Typhimurium 14028s genome coordinates 3005740–3050161 (Genbank ID CP001363.1) in each parent strain was replaced by a chloramphenicol resistance cassette flanked by FRT recombinase sites from plasmid pKD3 using primers ALO76 and ALO77. Each knockout mutation was then transduced into a fresh strain background by P22 HT105/1 *int*-201 transduction. Similarly, the SPI-2 deletion mutants were generated using the lambda red recombinase method. A chloramphenicol resistance cassette was amplified from plasmid pKD3 with primers ALO83 and ALO84, which were designed with flanking sequences complementary to the SPI-2 region. Following lambda red recombinase with the amplified PCR product, a 25 kb SPI-2 deletion spanning nucleotides 1,486,143–1,511,465 (Genbank ID CP001363.1) was introduced into the *S.* Typhimurium 14028s genome. The SPI-2 mutation was then transduced into a fresh strain background by P22 HT105/1 *int*-201 transduction. The double ΔSPI-1/ΔSPI-2 mutants strain were generated by first flipping the ΔSPI-1 chloramphenicol resistance cassette out of the chromosome by introducing the plasmid PCP20 expressing the FLP recombinase, and then introducing the SPI-2 deletion via P22 transduction.

Strains containing the *hilD* mutants were constructed by P22 transduction of a previously constructed mutation provided generously by the lab of Dr. Ferric Fang at the University of Washington [97].

### Experimental evolution of *Salmonella hns* mutants

An *hns* gene knockout from *S.* Typhimurium 14028s harboring a kanamycin resistance cassette in place of *hns* was moved into a fresh *S.* Typhimurium 14028s background containing a mutated *rpoS* allele (rpoS*) via P22 phage transduction. The transductants were selected on LB-agar plates supplemented with 50 µg/ml kanamycin. Six independently derived colonies from the original transduction were streaked twice on LB-kanamycin plates to eliminate trace P22 phage lysate, with each passage on solid media corresponding to a 16 hour incubation period at 37°C. All six transductants harbored the kanamycin resistance cassette in place of *hns* and were free of contaminating P22 phage as determined by PCR. These *hns* mutant isolates were selected to inoculate 5 ml LB in conical 25 ml polypropylene culture tubes. The cultures were grown at 37°C with 200 rpm shaking and every 24 hr, 5 µl from each culture was transferred to 5 ml of fresh media. The 1∶1000 dilution corresponds to approximately 9.96 doublings a day for a total of ∼300 doublings over the course of the 30 day evolution period. Daily samples from each lineage were taken and stored at −80°C in culture media supplemented with 10% DMSO for later analysis.

### Preparation of genomic libraries for Illumina sequencing

Samples from the frozen DMSO stocks representing day 1 and day 30 of the evolution period were scraped into LB media and grown at 37°C with shaking until mid-stationary phase (approximately 8 hours). The genomic DNA from approximately 4×10^9^ cells from each culture was purified using the Qiagen DNeasy blood and tissue kit. 5 µg of the purified DNA in 130 µl water was sheared to a mean fragment size of 400 nt using a Covaris S2 focused ultrasonicator (Woburn, Massachusetts). The fragmented DNA was concentrated in a centrifugal evaporator to less than 34 µl and treated with the End-IT DNA repair kit from Epicenter to blunt-end the DNA. Following a 1 hr incubation period at room temperature, 50 µl H_2_0 and 400 µl buffer QG from the Qiaquick Gel extraction kit were added to the blunted DNA fragments. The samples were purified with the QIAquick spin columns (Qiagen) and eluted twice in 15 µl elution buffer (total elution volume 30 µl). A-tails were added to the blunted fragments using the Klenow Exo-minus enzyme from Lucigen for 1 hr at room temperature and the reaction was terminated with the addition of 400 µl Quiagen QG buffer. After a second purification with the QIAquick spin columns, the eluted DNA (30 µl) was reduced to a volume of 9.25 µl in the centrifugal evaporator. Preannealed dsDNA adapter oligonucleotides were ligated to each sample overnight at 16°C using the Fast-Link DNA Ligation kit (Epicentre). These adapters were generated by mixing equimolar parts of a desalted common oligonucleotide (5′-AAT GAT ACG GCG ACC ACC GAG ATCTAC ACT CTTTCC CTA CAC GAC GCT CTT CCG ATC*T-3′), where C* indicates the addition of a phosphothioate group, and a unique indexing oligonucleotide with partial complementarity (5′Phos-GATCGGAAGAGCGGTTCAGCAGGAATGCCGAGACCGNNNNNNNNATCTCGTATGCCGTCTTCTGCTTG-3′), where N indicates a unique 8 nt barcode. The samples were separated on a 2% agarose gel and a slice containing fragments of approximately 400–450 nucleotides was extracted purified with the Qiagen gel extraction kit. The samples were then amplified in a PCR cycler for 16 cycles, purified once again with the Qiagen Gel Extraction Kit, and were quantified spectrophotometrically. Equal quantities of each library were combined and sequenced by the Donnelly Sequencing Centre (Toronto) in a partial lane of a 130 nt×8 nt index×100 nt paired-end run on an Illumina HiSeq2000 instrument using v3 chemistry. To achieve greater depth of coverage for the wild type E lineage at 30 days, this library was resequenced on a partial lane of a 101 nt×8 nt×101 nt HiSeq2500 run. The unique 8 nt barcode sequence present on the ligated adapters enabled the identification of each sample during downstream analysis.

### Tracking genetic changes in the *hns* mutant lineages

Paired-end Illumina reads from each strain were reference assembled to the published *S.* Typhimurium 14028s genome (Genbank ID CP001363.1) using the Geneious Pro 5.5.6 software package on “medium-low sensitivity/fast”, which corresponds to the following settings: maximum gaps per read 10%, maximum gap size 15, minimum overlap identity 80%, minimum overlap 25 nt, and maximum mismatches per read 20%. The mean genomic depth of coverage ranged from 32.2%–134.2%. Single nucleotide polymorphisms and small deletions and insertions (SNPs/INDELS) that arose in each lineage were identified by comparing the genomes of each lineage at Day 30 to their corresponding genome sequence at Day 1 using the Geneious Pro 5.5.6 “Find Variants/SNPs” tool with the minimum depth of sequence coverage 25-fold and the variant frequency set to 0.8. In addition, the raw Illumina reads of the *hns* mutant lineage C genomic DNA from Day 30 were aligned to the published 14028s genome using Bowtie version 1.0.0 and *de novo* assembled using Velvet version 1.2.1.0 [67], [68]. A list of the SNPs/INDELS from the Bowtie and Velvet assemblies of *hns* lineage C was generated using Samtools [69].

SNPs were then confirmed by sequencing PCR products from each strain. For each SNP, the corresponding gene was amplified from the DMSO stock of each passage by PCR using gene-specific primers: *stpA* (ALO117/118), *rpoD* (ALO122/123), *idnK* (ALO139/140), *mutY* (ALO141/142), *phoP* (ALO145/146), *phoQ* (ALO143/144), *yecS* (ALO151/152), *yhfC* (153/154) (Table 4). The resultant PCR product was then purified using EZ-10 Spin Column PCR Purification Kit (Biobasic) and sent for Sanger Sequencing at TCAG Sequencing Facility (Centre for Applied Genomics, Hospital for Sick Children). The passage when each mutation occurred was similarly determined by sequencing individual loci from the samples stored daily during the course of the experiment. Sequencing chromatograms were visually compared for the emergence of the mutant nucleotide change. Emergence of the SPI-1 deletions were detected by PCR amplifying the region spanning the deletion sites from the DMSO stock of each passage using the following primer pairs: ALO127/ALO128 for *Δhns* lineage A, ALO129/ALO130 for *Δhns* lineage B, ALO131/ALO132 for *Δhns* lineage D, ALO133/ALO134 for *Δhns* lineage E and ALO135/ALO136 for *Δhns* lineage F. The PCR products were purified using EZ-10 Spin Column PCR Purification Kit (Biobasic) and sent for Sanger Sequencing at TCAG Sequencing Facility (Centre for Applied Genomics, Hospital for Sick Children).

### Growth assays

Overnight cultures (5 ml LB) were inoculated from single colonies grown for approximately 16 hours at 37°C with 200 rpm shaking. Cultures for each strain were then adjusted to an O.D. at 600 nm of 0.5 and then diluted an additional 1∶100. 200 µl of each culture was then dispensed in triplicate into a clear, flat-bottom 96-well plate (Sarstedt), the plate was covered with the plate lid and grown overnight with shaking at 37°C in a TECAN Infinite M200 Pro microplate reader. Optical density readings at 600 nm were recorded every 15 minutes for 18 hours.

### Motility assays

Overnight cultures (5 ml LB) were initiated from single colonies and grown for 16 hour at 37°C with shaking at 200 rpm. The next day cultures were each adjusted by dilution to an O.D. at 600 nm of 0.1. Equivalent colony forming units in each of the diluted cultures were verified by plating serial dilutions. 5 µl of the O.D._600nm_ 0.1 cultures was spotted into the center of 25 ml soft agar plates (LB 0.35% agar). The plates were incubated for 12 hours at 37°C and the radial swarming diameters were measured. The motility assays were replicated three times and in each assay the strains were plated in triplicate.

### Immunoblot assays

Overnight cultures were diluted 1∶200 in 200 ml LB media containing 20 µg/ml chloramphenicol. Sample volumes of 50 ml, 12 ml and 1.5 ml were removed from the cultures at O.D. 600 nm of 0.1, 0.6 and 1.5 respectively. Cells were harvested by centrifugation at 5000× g for 15 min at 4°C. The cell pellets were resuspended in cell lysis buffer containing 9.32 M urea, 2.67 M thiourea, 40 mM Tris, and 86.78 mM 3-(3- cholamidopropyl)-dimethylammonio-1-propanesulfonate (CHAPS; pH 8.5). Cells were lysed by sonication and the total protein concentrations were quantified using Bradford assay (Bio-Rad). 30 µg of total protein was combined with 2× SDS PAGE loading dye and separated on a 16% polyacrylamide SDS Tris-Tricine gel. Transfer to a nitrocellulose membrane was performed with the Bio-Rad semidry electrophoretic transfer cell at 15 V for 1 h. The membrane was blocked at 4°C over night in TBST 1× Tris-buffered saline, 0.05% Tween 20) with 5% skim milk powder. The membrane was probed with Rabbit anti-FLAG M2 antibody (Sigma) diluted 1∶1000 in TBST with 5% (w/v) skim milk for 1 h at room temperature, followed by goat anti-rabbit secondary antibody conjugated with horseradish peroxidase (Sigma, diluted 1∶10,000 in TBST with 5% milk) for 1 h at room temperature. DnaK was probed as a loading control using a mouse primary antibody (Enzo Life Sciences, 1∶1,000 in TBST with 5% milk) followed by a goat anti-mouse secondary antibody conjugated with horseradish peroxidase (Enzo Life Sciences, 1∶10,000 in TBST with 5% milk).

### Protein expression and purification

Constructs overexpressing StpA_wt_, StpA_T37I_, StpA_M4T_ and StpA_A77D_ were transformed in BL21Δ*hns* (DE3) strain. The resulting strains were cultured in Luria Bertani (LB) until OD_600 nm_ = 0.6. IPTG was added to a final concentration of 1 mM prior to growing the cultures for 16 h at 18°C. Cells were spun at 2500×g for 30 min, resuspended in 20 mL cell lysis buffer (20 mM Tris pH 8, 500 mM NaCl, 5 mM imidazole, 5 mM β-mercaptoethanol) and sonicated. The cellular debris was removed by centrifugation at 20 000 g for 15 min. Ni^2+^ resins were incubated with supernatant for 1 h on a rocking platform, washed twice with washing buffer (20 mM Tris pH 8, 500 mM NaCl, 30 mM imidazole, 5 mM β-mercaptoethanol), and eluted with elution buffer (20 mM Tris pH 8, 500 mM NaCl, 500 mM imidazole). Proteins were then purified further by size exclusion chromatography using Superdex 200 16/60 column from GE healthcare using storage buffer (20 mM Tris pH 8, 1 M NaCl, 1 mM EDTA and 5% glycerol). Fractions containing protein were concentrated using Millipore Amicon Ultra centrifugal filter 3K and stored at −80°C. H-NS_6HIS_ protein was purified by nickel affinity chromatography as previously described [98]. Ni^2+^ purified H-NS_6HIS_ was dialyzed in buffer A (20 mM Tris pH 8, 1 mM EDTA, 200 mM NaCl and 5% glycerol) overnight prior to being loaded onto a 5 mL Hitrap Heparin HP column, and eluted using a linear gradient of low salt buffer (20 mM Tris pH 8, 1 mM EDTA, 150 mM NaCl and 5% glycerol) with high salt buffer (20 mM Tris pH 8, 1 mM EDTA, 1 M NaCl and 5% glycerol) over 120 mL. Peak fractions were analyzed by SDS-PAGE and then dialyzed in loading buffer prior to storage at −80°C.

### Transcript analysis

Total RNA was purified and reverse transcribed as previously described [98]. The resulting cDNA was analyzed by real-time quantitative PCR (Q-PCR) with primers specific to *ssrA* (SSA198/199), *hilA* (SSA200/201), *proV* (SSA202/203) and *yciG* (SSA232/233). *gyrB* served as an internal control for normalization and was analyzed with primer set WNp233/234. Q-PCR was performed with the SsoFast Evagreen Supermix (Bio-Rad) according to the manufacturer's instructions.

### Electrophoretic mobility shift assay (EMSA)

Two DNA fragments were employed in this assay; a 289 bp fragment of *hilA* (%GC = 34) from *S.* Typhimurium 14028s genomic DNA, and a 204 bp GC-rich fragment of PA3900 (%GC = 74) from *Pseudomonas aeruginosa* strain PAO1. The *hilA* fragment was amplified by PCR using primers GT068 and GT077 and the PA3900 fragment was amplified using primers GT049 and GT050 (Table 5). Various concentrations of purified H-NS, StpA_WT_ and relevant variants of StpA were incubated with 10 nM DNA in binding buffer (15 mM HEPES pH 7.9, 40 mM KCl, 1 mM EDTA, 0.5% DTT, 5% glycerol) for 30 minutes. 4 µl of 6× Fermentas loading dye was added to each 20 µl reaction immediately prior to separation by gel electrophoresis for 2.5 h at 70 V on a 6% native polyacrylamide gel at 4°C (buffered with Tris acetate EDTA). Gels were stained with SYBR Green for 20 minutes at room temperature, washed twice with ddH_2_O, and DNA complexes were visualized with ultraviolet light.

## Supporting Information

Figure S1 **Location of the SPI-1 genomic deletions.** The SPI-1 deletion sites were PCR amplified from the day 30 frozen culture stocks and the resulting PCR products were sequenced. The nucleotides adjacent to the SPI-1 deletion sites that were deleted from the evolved *hns* mutant lineages are represented in grey font. The four nucleotides flanking either side of the deletion sites are highlighted in orange. Blue was used in place of orange where the flanking nucleotides are direct repeats. The nucleotide positions of the deletion sites in the *S.* Typhimurium 14028S reference genome are indicated with darts above each sequence.
(TIF)

Figure S2 **Disruption of SPI-2 modestly improves fitness of an** ***hns*** **mutant.** A SPI-2 deletion spanning nucleotides 1,486,143 to 1,511,465 in the *S.* Typhimurium 14028s genome (Genbank ID CP001363.1) was introduced into a *Δhns* and a *Δhns/ΔSPI-1* background. Growth of the *Δhns/ΔSPI-2* (orange curve) and *Δhns/ΔSPI-1/ΔSPI-2* (green curve) mutants was monitored in a 96 well plate reader alongside wild type *S.* Typhimurium (black curve), a *Δhns* strain (red curve) and the *Δhns/ΔSPI-1* mutant (blue curve). Plotted is the average of three biological replicates and standard error.
(TIF)
