# Supplementary figures and images for: Silencing by H-NS Potentiated the Evolution of Salmonella
Source: PLoS Pathog. 2014 Nov 6;10(11):e1004500. doi: 10.1371/journal.ppat.1004500 (PMC4223078; doi:10.1371/journal.ppat.1004500)

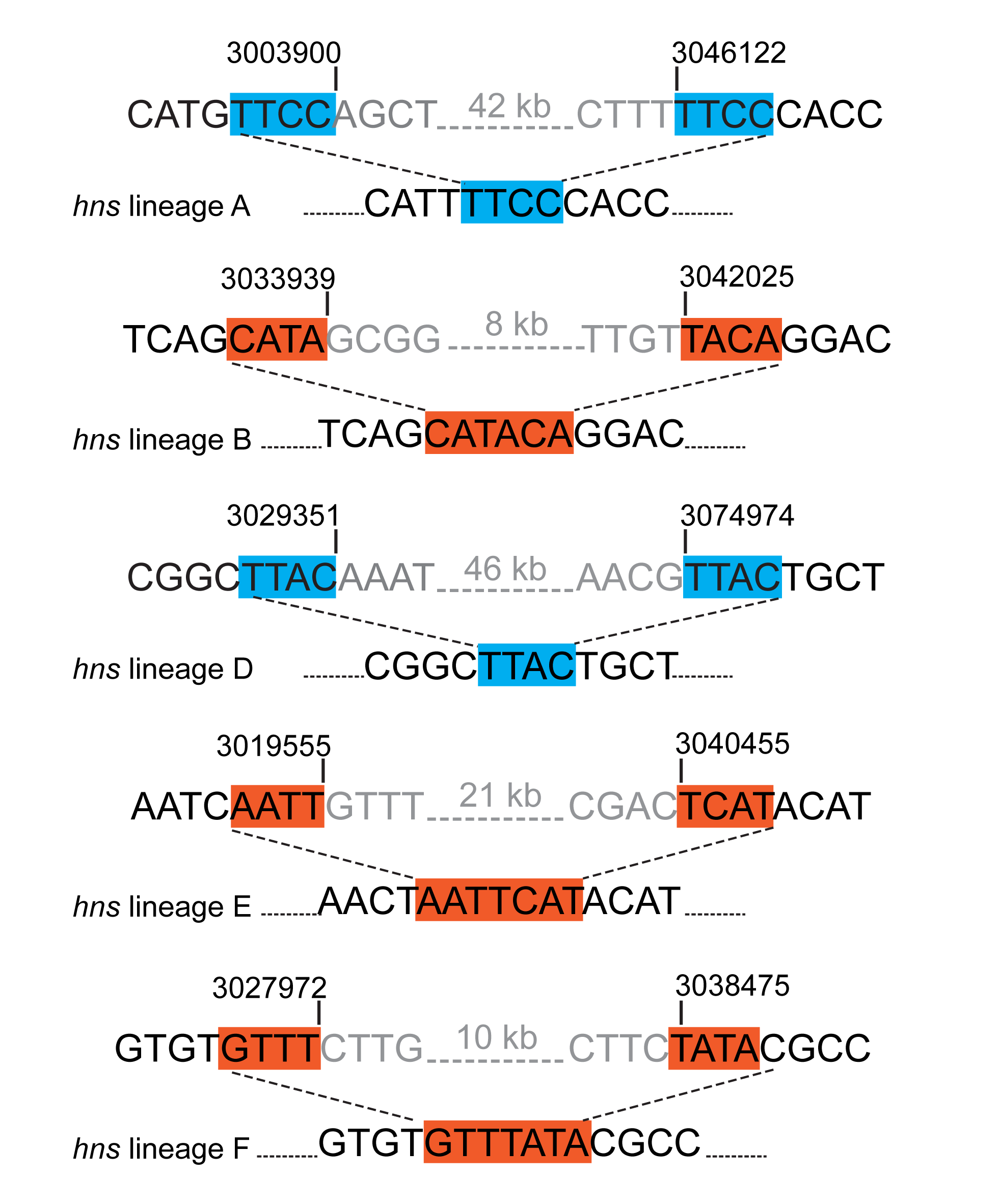

Supplement: Figure S1 — Location of the SPI-1 genomic deletions. The SPI-1 deletion sites were PCR amplified from the day 30 frozen culture stocks and the resulting PCR products were sequenced. The nucleotides adjacent to the SPI-1 deletion sites that were deleted from the evolved hns mutant lineages are represented in grey font. The four nucleotides flanking either side of the deletion sites are highlighted in orange. Blue was used in place of orange where the flanking nucleotides are direct repeats. The nucleotide positions of the deletion sites in the S. Typhimurium 14028S reference genome are indicated with darts above each sequence. (TIF) [file ppat.1004500.s001.tif]

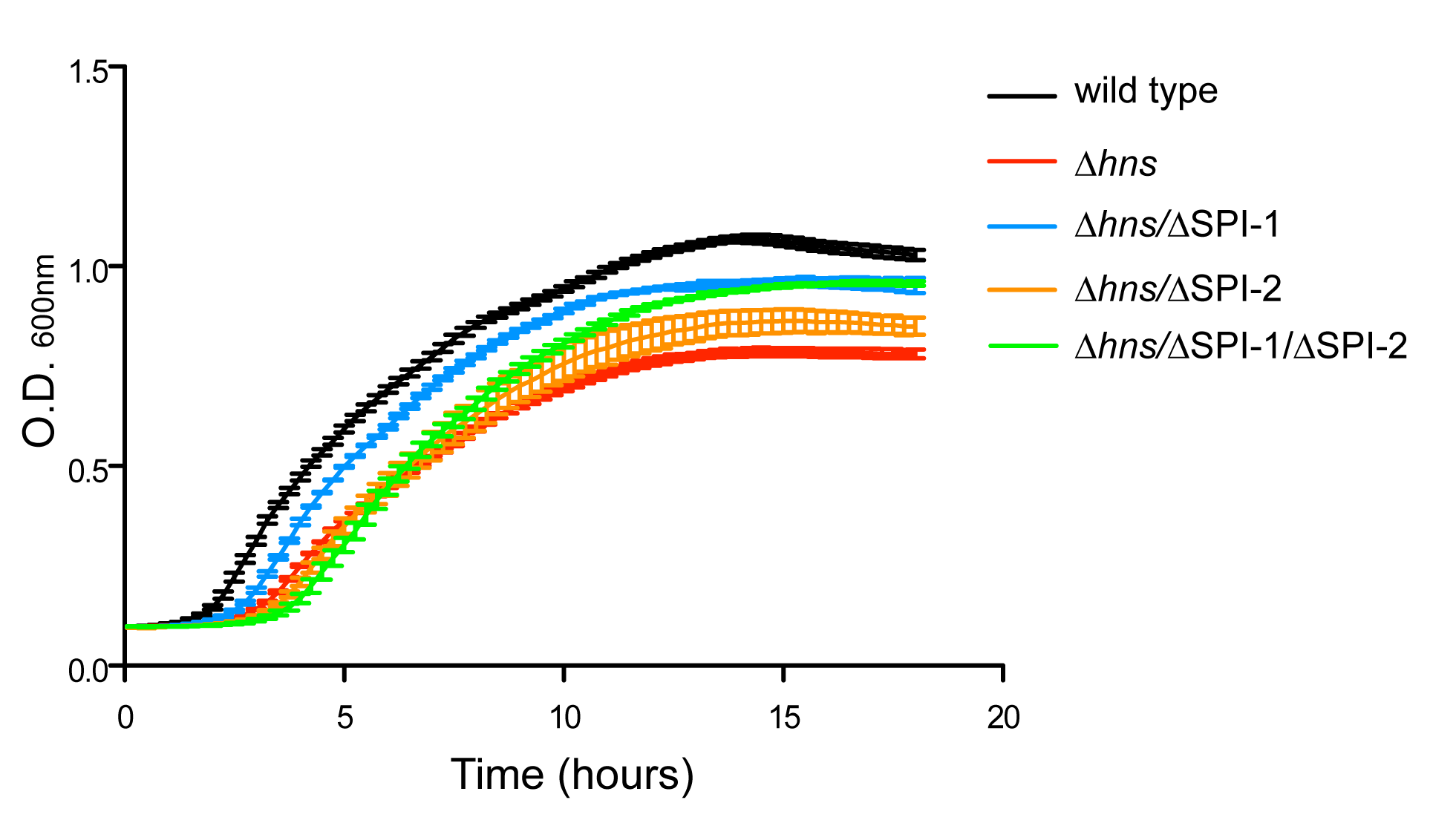

Supplement: Figure S2 — Disruption of SPI-2 modestly improves fitness of an hns mutant. A SPI-2 deletion spanning nucleotides 1,486,143 to 1,511,465 in the S. Typhimurium 14028s genome (Genbank ID CP001363.1) was introduced into a Δhns and a Δhns/ΔSPI-1 background. Growth of the Δhns/ΔSPI-2 (orange curve) and Δhns/ΔSPI-1/ΔSPI-2 (green curve) mutants was monitored in a 96 well plate reader alongside wild type S. Typhimurium (black curve), a Δhns strain (red curve) and the Δhns/ΔSPI-1 mutant (blue curve). Plotted is the average of three biological replicates and standard error. (TIF) [file ppat.1004500.s002.tif]
